# Taxonomic diversity of terrestrial vertebrates in west-central Mexico: Conservation from a multi-taxa perspective

**DOI:** 10.1371/journal.pone.0311770

**Published:** 2024-10-09

**Authors:** Eliza Álvarez-Grzybowska, Verónica Carolina Rosas-Espinoza, Karen Elizabeth Peña-Joya, Ana Luisa Santiago-Pérez, Luis Ignacio Íñiguez-Dávalos, Miguel Ángel Macías-Rodríguez, Fabián Alejandro Rodríguez-Zaragoza

**Affiliations:** 1 Doctorado en Biosistemática, Ecología y Manejo de Recursos Naturales y Agrícolas (BEMARENA), Centro Universitario de Ciencias Biológicas y Agropecuarias, Universidad de Guadalajara, Zapopan, Jalisco, México; 2 Departamento de Ecología Aplicada, Laboratorio ds1e Ecología Molecular, Microbiología y Taxonomía (LEMITAX), Centro Universitario de Ciencias Biológicas y Agropecuarias, Universidad de Guadalajara, Zapopan, Jalisco, México; 3 Laboratorio de Ecología, Paisaje y Sociedad, Centro Universitario de la Costa, Universidad de Guadalajara, Puerto Vallarta, Jalisco, México; 4 Departamento de Producción Forestal, Centro Universitario de Ciencias Biológicas y Agropecuarias, Universidad de Guadalajara, Zapopan, Jalisco, México; 5 Departamento de Ecología y Recursos Naturales, Centro Universitario de la Costa Sur, Universidad de Guadalajara, Autlán de Navarro, Jalisco, México; 6 Departamento de Ciencias Ambientales, Centro Universitario de Ciencias Biológicas y Agropecuarias, Universidad de Guadalajara, Zapopan, Jalisco, México; Southeastern Louisiana University, UNITED STATES OF AMERICA

## Abstract

Multi-taxa approaches are increasingly used because they describe complementary aspects of ecosystem dynamics from a community ecology perspective. In west-central Mexico, the complex biogeography and topography have created an environment where temperate and tropical forests converge, resulting in great biological diversity. Within this region, the Sierra de Quila Natural Protected Area (SQPA) offers an important example for understanding ecological community dynamics. We analyze the taxonomic diversity of terrestrial vertebrates in the SQPA by incorporating taxonomic levels associated with species. We evaluated the taxonomic diversity with i) an average taxonomic distinctiveness analysis (alpha diversity) and ii) an analysis of taxonomic dissimilarity and partitioning of turnover and differences in richness components (beta diversity). Tropical forests boast the highest taxonomic diversity of amphibians, reptiles, and birds, while temperate gallery forests exhibit lower values. Our results showed that terrestrial vertebrate alpha and beta diversity patterns respond mainly to contrasting vegetation types (tropical *vs*. temperate). Regarding beta diversity, the multi-vegetation type analysis showed the highest values for reptiles, followed by amphibians, birds, and mammals. Turnover had the highest contribution to beta diversity, while differences in richness were relevant for amphibians and reptiles, which could be related to their low mobility and sensitivity to environmental conditions. Despite the local scale, the SQPA presented high beta diversity, reflecting historical ecological processes in taxonomic composition derived from contrasting environments and constraints imposed on species. Evaluating taxonomic structure from a multi-taxa perspective is essential for conservation efforts because it allows the spatial recognition of biological assemblages as a first step for local interventions.

## 1 Introduction

In the face of ongoing biodiversity loss, natural protected areas (NPAs) have become the main tool for wildlife preservation [1–4]. In Mexico, they occupy 13% of forested areas [5,6]. However, NPAs’ effectiveness has been questioned because they only consider a few biological groups and the keystone or charismatic species for management, thus ignoring the pool of regional species that are part of local diversity [3,7]. This results in poorly known or difficult-to-detect species being poorly represented [8,9]. Multi-taxa perspectives are increasingly used because they provide complementary insights into ecosystem dynamics from a community ecology standpoint [10–12]. In terrestrial ecosystems, vertebrates are suitable for these approaches due to their wide distribution, mobility, and physiological tolerances, which allow them to inhabit various environments [13–16].

Biodiversity is defined as the variety of organisms in a location and the ecological complexity in which they live. Alpha and beta diversity describe biodiversity patterns across space and time [17–19]. Alpha diversity evaluates community structure by examining species richness and taxonomic distinctiveness at the local scale [18,20]. Conversely, beta diversity assesses the response of organisms to spatial gradients. It is relevant for explaining differentiation patterns at the regional level [17,21,22].

Taxonomic diversity has been proposed as a tool for monitoring and tracking conservation policies because it is not affected by scale [23]. Alpha and beta diversity can be estimated by incorporating taxonomic levels associated with species (i.e., genus, family, and order), providing information on their ecological and evolutionary history as a proxy for phylogenetic diversity [20,24]. This approach is practical when incorporating diverse biological groups with little or no information about their phylogenetic past into the analysis [25]. Taxonomic distinctiveness represents the alpha component because it measures the degree of taxonomic relatedness among species in each sample as a reflection of the ecological and evolutionary mechanisms that determine taxonomic composition [20,26]. In contrast, each biological group contributes to beta diversity patterns in a different and complementary way due to their response to environmental constraints [15,27]. Beta diversity can be partitioned into turnover (species replacement) and differences in richness components (species loss or gain) [26,28]. In this way, the incorporated taxonomic levels become elements that provide a more realistic (and not overestimated) view of taxonomic beta diversity [10].

The Mexican Transition Zone in west-central Mexico is distinguished by its topographic complexity and biotic mix (Nearctic-Neotropical), which is responsible for the abrupt coexistence between temperate and tropical forests [29]. This heterogeneity has created high diversity and endemism resulting from its geological and climatic history [30–32]. For this reason, west-central Mexico is considered a biodiversity hotspot within Mesoamerica [12,32–34]. Vertebrates show different occurrence patterns between temperate and tropical environments in response to environmental heterogeneity (topographic complexity) and biotic mix. For these organisms, turnover has become important for revealing *in situ* speciation and extinction processes [12,27,35]. Assessing diversity patterns (alpha and beta) of terrestrial vertebrates at large scales has helped set conservation priorities [12,36–38].

The Sierra de Quila Protection Area (SQPA) is a mountain with temperate and tropical forest areas. It shelters a representative sample of the vertebrate diversity in west-central Mexico [39]; thus, it is a good model for understanding the dynamics of ecological communities in a highly heterogeneous region. This study aimed to analyze the taxonomic diversity of terrestrial vertebrates in different vegetation types in the SQPA from a multi-taxa perspective. Taxonomic diversity was assessed using the following approach: i) Alpha diversity was calculated with an average taxonomic distinctiveness analysis, and ii) Beta diversity was calculated with a taxonomic dissimilarity assessment and partitioning its components of turnover and differences in richness. We hypothesized that: i) Due to the heterogeneity of tropical areas, tropical deciduous forest and tropical gallery forest would show a higher taxonomic diversity of vertebrates; ii) The highest taxonomic dissimilarity would be found between temperate and tropical vegetation types, where turnover is the major contributor to taxonomic differentiation due to the contrasting characteristics and environmental constraints imposed by the vegetation types.

## 2 Materials and methods

### 2.1 Study area

**Fig 1:**
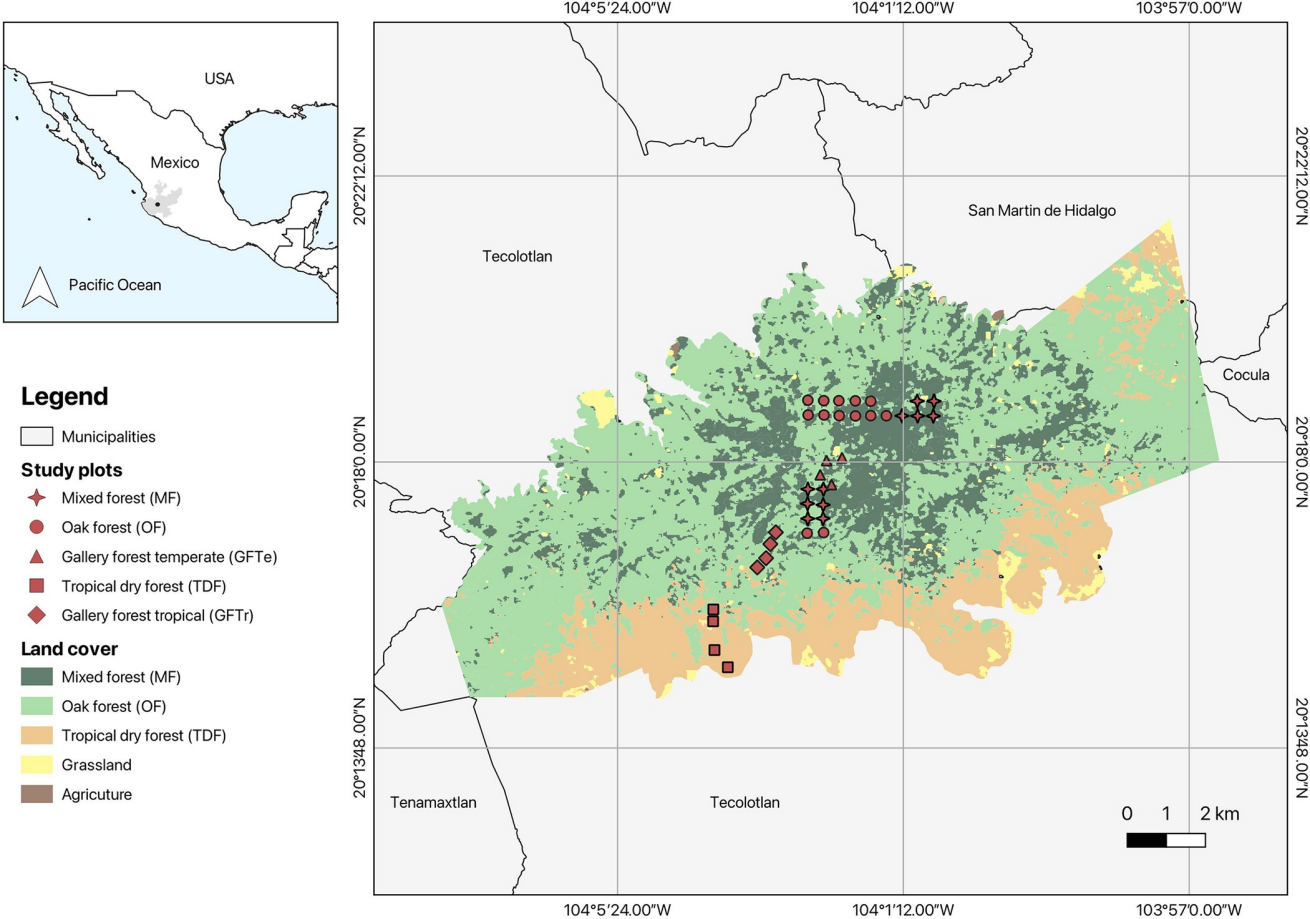
The study area shows the coverage of vegetation types and the location of 38 sampling points in the SQPA, Mexico. The map was created by E.A.G. QGIS 3.30.3 software (https://www.qgis.org/es/site/).

This study was conducted in the SQPA (20°22’20.14’ N, 104°09’103.57’ W) within the municipalities of Tecolotlán, San Martín de Hidalgo, Tenamaxtlán, and Cocula, in the state of Jalisco, Mexico (Fig 1). This area is part of the Mexican Transition Zone and has an elevation range from 1,350 to 2,560 m asl [6].

The sampling area presents two contrasting environments with summer rainfall (882 mm of mean annual precipitation): i) Temperate climate (Cw) (1,500–2,560 m a.s.l.), a sub-humid temperate climate with a mean annual temperature of 12°C to 18°C; and ii) Tropical climate (Aw) (1,200–1,600 m a.s.l.), humid warm climate with a mean annual temperature higher than 18°C [40,41]. The SQPA covers an area of 15,192 ha and features mountainous relief with small valleys covered by different vegetation types [6]:

### Tropical area

1. **Tropical dry forest (TDF)** (14.4% of the total area). This vegetation type is found around the protected area in the lower zones between 1,200 and 1,700 m asl. *Quercus resinosa*, *Acacia pennatula*, *Bursera* spp., *Eysenhardtia polystachya*, and *Ipomea murucoides* are some of the most dominant species.
2. **Tropical gallery forest (GFTr).** This vegetation type grows along streams between 1,200 and 1,700 m asl. Species such as *Salix bonplandiana*, *Phoebe psychotrioides*, *Alnus acuminata*, *Ficus goldmanii*, and *Xylosoma velutinum* are present.

### Temperate area

3. **Oak forest (OF)** (17% of the total area). It occurs between 1,500 and 1,900 m asl. *Q*. *resinosa*, *Pinus oocarpa*, and *Pinus douglasiana* are the most dominant species.
4. **Mixed forest (MF)** (56.7% of the total area). It is distributed between 1,800 and 2,520 m asl. Dominant forest species include *Pinus lumholtzii*, *P*. *douglasiana*, and *Q*. *resinosa*.
5. **Temperate gallery forest (GFTe).** This vegetation type extends along streams and is characterized by some elements of mountain mesophytic forest. It is distributed between 1,800 and 2,520 m asl. The most common species are *Alnus acuminata*, *Clethra hartwegii*, *Pinus devoniana*, *Prunus serotina*, *Styrax ramirezii*, and *S*. *bonplandiana*.

GFTe and GFTr had few vegetation patches in temperate and tropical areas; the remaining cover (3.5%) corresponds to agriculture and pasture areas [40,41]. During the study, we obtained access from private owners of land in the natural area to carry out the sampling.

### 2.2 Vertebrate sampling

For amphibian, reptile, and bird sampling, 38-point counts were established for the observation and counting of organisms (500 m^2^ for amphibians and reptiles, and 1,257 m^2^ for birds), spaced at least 250 m from the edge of each point count [42]. We visited each point count monthly and carried out specific identification of the observed organisms from January 2009 to June 2010. The number of points was determined based on the percentage of the surface area occupied by vegetation type in the NPA, according to the map generated by Villavicencio [40], and the respective sites’ accessibility: 13 in OF, 11 in MF, six in TDF, four in GFTe, and four in GFTr. Two expert teams were formed for taxonomic groups to avoid bias in records during simultaneous sampling: one for amphibians and reptiles and another for birds. Once a month, each team, consisting of two people, sampled birds at each counting point for 10 minutes and sampled amphibians and reptiles for 20 minutes. For amphibians and reptiles, an intensive unrestricted search was conducted in the microhabitats preferred by these species at each point count (e.g., logs and rocks) [43]. For birds, organisms were identified by observation and auditory recognition at each count point [44]. Each team sampled vegetation types on different days to avoid overlap. Additionally, nocturnal sampling (sampling conducted between 21:00–23:00 hours) was conducted in 2,400 m^2^ transects to detect nocturnal amphibian and reptile species in each vegetation type.

For mammals, twelve point counts were established in the vegetation types: two in TDF, three in OF, and seven in MF. Ten surveys were conducted at all point counts during 2009; direct observations, tracks, and scats were recorded for medium and large mammals [45]. For small mammals, 40 Sherman traps arranged in four transects across all vegetation types were used during six surveys conducted between January and May 2009. For bats, 14 nocturnal surveys were performed between April 2011 and May 2012, with each point count visited at three-month intervals to sample all twelve sites in each climatic season. Three large mist nets (12 x 3 m) and three medium mist nests (8 x 3 m) were placed 30 m apart and observed every 30 minutes for four hours after sunset [46].

Species were determined using specialized literature on amphibians and reptiles [39], birds [47,48], and mammals [13,45,49,50]. According to the IUCN Red List of Threatened Species and NOM-059-SEMARNAT-2010 (Mexican government regulation), no wild or threatened species were collected during sampling to remove them from the SQPA [51,52]. Photographs of the species were deposited in the Zoology Museum "Alfonso L. Herrera" of the Universidad Nacional Autónoma de México (UNAM).

### 2.3 Data analysis

To explore spatial differences in alpha and beta diversity of terrestrial vertebrate taxonomic structure, we used a one-way experimental design with vegetation type as a factor with five levels (MF, OF, TDF, GFTe, GFTr).

#### 2.3.1 Alpha taxonomic diversity

To assess the variation of species and higher taxa in the different vegetation types, we estimated average taxonomic distinctiveness (Δ^+^) and its variance (Λ^+^) for all biological groups (overall) and by taxonomic groups for each vegetation type. These metrics measure the average taxonomic length between two randomly chosen species and its variance, according to the equations of Warwick and Clarke [20] (Eqs 1 and 2):

$$\Delta^{+}=[\sum \sum_{i<j}\omega_{ij}]/[s(s-1)/2]$$ (1)

$$\Lambda^{+}=[\sum \sum_{i\neq j}\omega_{ij-\omega }2]/[s(s-1)/]$$ (2)

Where *s* represents the number of species, and *ω*_*ij*_ indicates the weight assigned to each taxonomic level. For these analyses, five taxonomic levels (species, genus, family, order, and class) were considered and weighted with a value *ω*_*ij*_ = 1 to easily identify those levels with the highest contribution to Δ^+^ and Λ^+^ [53]. Models were generated with a ratio of 1.2 species, both overall and for each biological group. Statistical significance of Δ^+^ and Λ^+^ was tested with 10,000 permutations (p ≤ 0.05) and represented by 95% confidence intervals (probability funnels). Analyses were performed with PRIMER 7.0.21 and PERMANOVA+ [52].

#### 2.3.2 Beta taxonomic diversity

Differentiation patterns in terrestrial vertebrate taxonomic structure among vegetation types were assessed with gamma taxonomic dissimilarity (Γ^+^; Eq 3). This measure represents the average of the taxonomic distances between species in one sample and their closest taxonomic relationship to species in another sample [53] as follows:

$$\Gamma^{+}=\frac{\left(\sum_{i=1}^{S1}minj\{\omega ij\}+\sum_{j=1}^{S2}mini\{\omega ij\}\right)}{\left(S1+S2\right)}$$ (3)

Where Γ^+^ expresses the gamma taxonomic dissimilarity, *S*_*1*_ and *S*_*2*_ represent the number of species in each sample, and *ω*_*ij*_ the taxonomic distances between species *i* and *j*. Subsequently, classification analyses were constructed from the dissimilarity matrices Γ^+^ among vegetation types (Q-mode) based on the UPGMA clustering method [54,55]. Finally, a similarity profile analysis (SIMPROF) with 10,000 permutations was performed for cluster identification. All analyses were performed with PRIMER v7 (7.0.21) and PERMANOVA+ [56]. Taxonomic beta diversity was also partitioned into the components of turnover (*β*.*3*) and differences in richness (*βrich*) [28] according to the following equations:

$$\beta_{CC}=1-\frac{b+c}{a+b+c}$$ (4)

$$\beta_{.3}=2x\frac{min(b,c)}{a+b+c}$$ (5)

$$\beta_{rich}=\frac{\left|b-c\right|}{a+b+c}$$ (6)

Where *β*_*CC*_ (4) represents the total dissimilarity (1 minus Jaccard’s similarity coefficient) divided into the components of dissimilarity due to turnover expressed as *β*_.*3*_ (5) and dissimilarity due to differences in richness expressed as *β*_*rich*_ (6). This beta diversity partitioning evaluated the taxonomic structure by considering all sites (multi beta) and comparing all vegetation types (paired beta). Therefore, we used the method proposed by Bacaro et al. [26], which considers total taxonomic beta diversity as *β*_*CC*_*T* = 1- Δ_*T*_ (7). This value expresses a total taxonomic dissimilarity between two assemblages considering the number of shared taxa among the total taxa. Values of *β*_*CC*_*T* are expressed from zero to one, where one represents total dissimilarity. For this analysis, five taxonomic levels (species, genus, family, order, and class) were considered for all groups, as per the following equation:

$$1-\Delta_{T}=\frac{{|G}_{AB}|}{\left|G_{A}|+|G_{B}|-|G_{AB}\right|}$$ (7)

Where |GA| and |GB| are defined as the number of taxa in the GA and GB taxonomic trees, respectively. Δ_*T*_ is equivalent to the number of shared taxa among the total taxa, and 1-Δ_*T*_ represents a normalized measure of taxonomic dissimilarity. Taxonomic beta diversity and partitioning of overall vertebrate components and by biological group were analyzed with the "BAT" package [57] and the script proposed by Carvalho et al. [28] and Diserud and Ødegaard [58]. Analyses and graphs were performed in R-project 4.1.1 [59].

## 3 Results

This work recorded 222 species of four classes, 22 orders, 66 families, and 150 genera, including 108 birds, 72 mammals, 24 reptiles, and 18 amphibians (S1 Table). The taxonomic levels with the most species were the order Passeriformes (birds), with 72 species; the family Vespertilionidae (mammals), with 15 species; and the genus *Sceloporus* (reptiles), with seven species. Twenty-eight percent of the species are endemic to Mexico, and 14% are in some risk category, according to NOM-SEMARNAT-2010 (Tables 1 and S2) [52]. TDF had the highest species richness of terrestrial vertebrates and taxonomic levels (genus, family, order, and class). In contrast, the highest species richness of amphibians, reptiles, and birds at the class level was found in GFTr, while the highest richness of mammals was recorded in MF (Table 1).

**Table 1:** Vertebrate species richness of the SQPA by vegetation type and taxonomic levels.

	TDF	GFTr			OF	MF		GFTe	Total
Class	4	4			4	4		4	4
Order	19	18			19	19		16	22
Family	52	49			47	47		40	66
Genera	100	99			90	93		76	150
Species	123	122			117	125		102	222
Biological groups						
Amphibians	5	13			5	4		5	18
Reptiles	8	11			11	11		2	24
Birds	59	62			53	54		50	108
Mammals	51	35			47	56		44	72

Codes: TDF, tropical dry forest; GFTr, tropical gallery forest; OF, oak forest; MF, mixed forest; GFTe, temperate gallery forest.

### 3.1 Alpha taxonomic diversity

**Fig 2:**
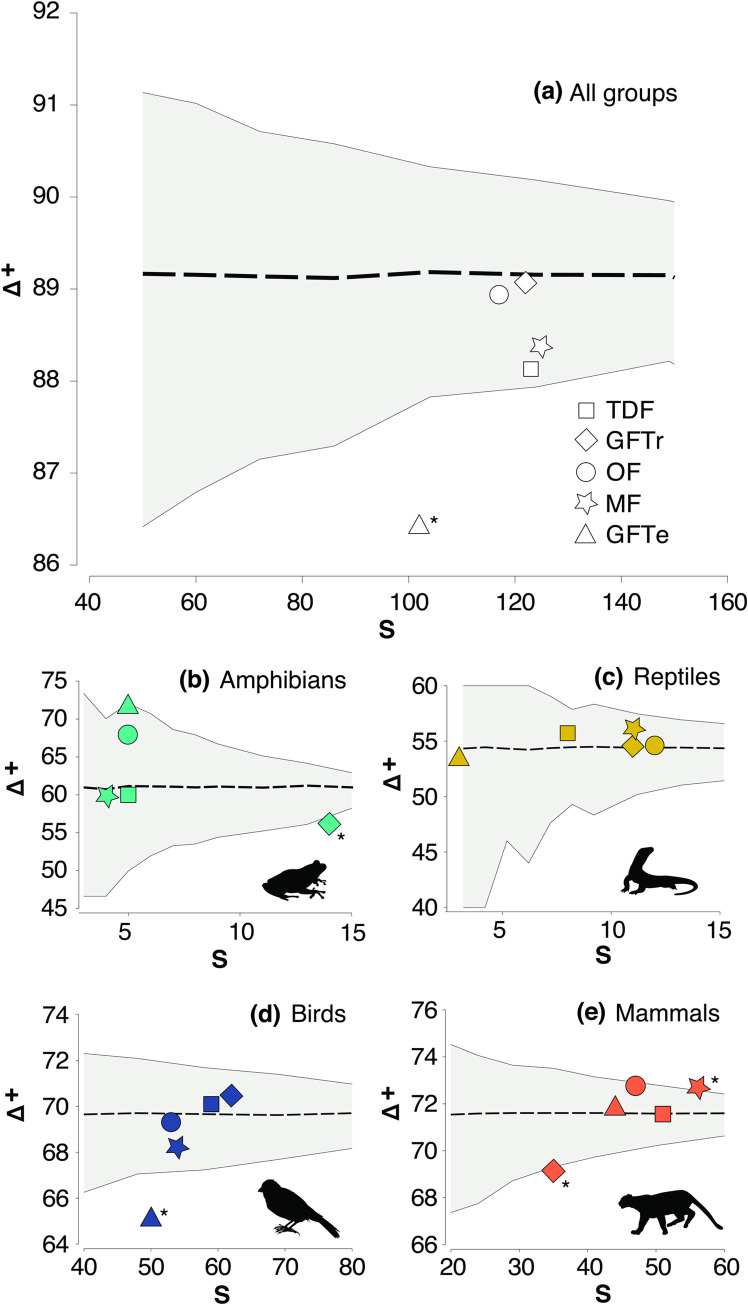
Analysis of average taxonomic distinctiveness (Δ^+^) among vegetation types: a) Overall vertebrate assessment; b) Amphibians; c) Reptiles; d) Birds; e) Mammals. The dotted line represents the average taxonomic distinctiveness, and the solid lines correspond to the lower and upper confidence intervals at the 95% level. Codes: S, species richness; TDF, tropical dry forest; GFTr, tropical gallery forest; OF, oak forest; MF, mixed forest; GFTe, temperate gallery forest. *Values outside the confidence intervals.

In the overall analysis of vertebrates, the highest average taxonomic distinctiveness (Δ^+^) was between order and class levels. All vegetation types were within 95% confidence intervals (p > 0.05), except GFTe (Δ^+^ = 86.4, p = 0.004), which had the lowest number of species. GFTr had the highest Δ^+^ value (Δ^+^ = 89.1) (Fig 2A; S4 Table). Similarly, the values of Λ^+^ variation for all vegetation types remained within the confidence limits (95%) expected from the model, indicating that the variation (Λ^+^) in Δ^+^ was as expected for all vegetation types, except for GFTe (Λ^+^ = 337.8, p = 0.002) (S4 Table).

Overall, family and order levels showed the highest Δ^+^ (between 60 and 80) in every biological group, except for reptiles, which had the highest distinctiveness between genus and family levels (between 40 and 60) (Fig 2; S4 Table). For amphibians, all Δ^+^ values by vegetation type fell within the probability funnel. However, despite being the vegetation type with the highest number of species, GFTr coincidentally had a lower Δ^+^ (Δ^+^ = 56.3) and Λ^+^ (Λ^+^ = 87.1) than expected (p = 0.05 and p = 0.016, respectively) (Fig 2B). For reptiles, all vegetation types remained within the probability funnel for both Δ^+^ and Λ^+^ (Fig 2C). For birds, the Δ^+^ values were within the probability funnel for all vegetation types except for GFTe (Δ^+^ = 65.1, p = 0.004), characterized by the lowest species richness. In contrast, for Λ^+^, vegetation types outside the model were OF (Λ^+^ = 183.7, p = 0.002) and MF (Λ^+^ = 180.6, p = 0.01) (Fig 2D). Finally, for mammals, the Δ^+^ values of GFTr (Δ^+^ = 69.1, p = 0.038) and MF (Δ^+^ = 72.7, p = 0.014) were outside the confidence intervals (95%) estimated by the model and had the lowest and highest number of species, respectively (Fig 2E; S4 Table).

### 3.2 Beta taxonomic diversity

**Fig 3:**
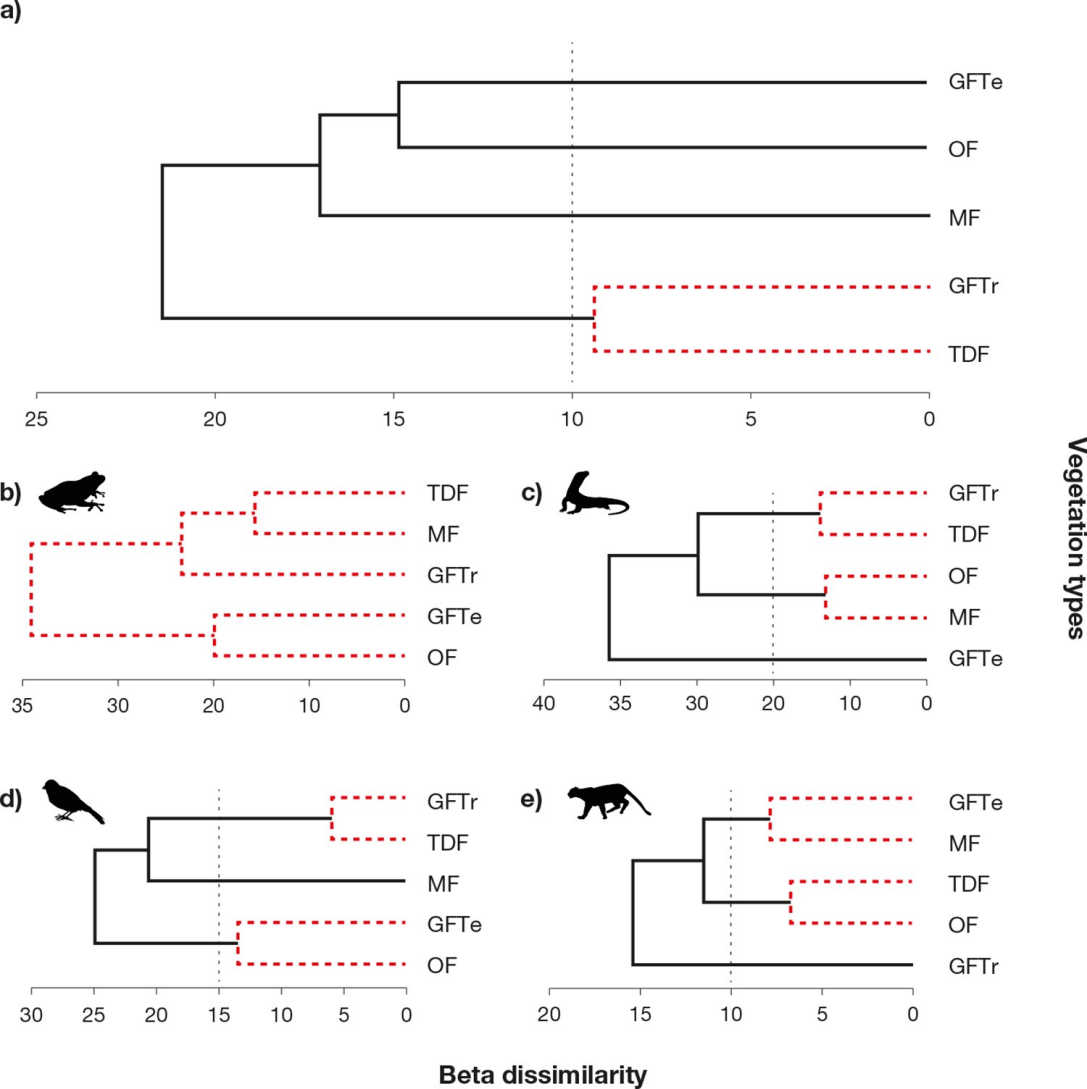
Beta taxonomic dissimilarity (cluster) of terrestrial vertebrates among vegetation types. Dissimilarity among vegetation types of vertebrates (a); amphibians (b); reptiles (c); birds (d); mammals (e). The cut-offs reflect the clusters identified by the similarity profile test (SIMPROF). Codes: TDF, tropical dry forest; GFTr, tropical gallery forest; OF, oak forest; MF, mixed forest; GFTe, temperate gallery forest.

In the classification analysis, the beta taxonomic dissimilarity of vertebrates showed low overall Γ^+^ values (max. 21.5%). The SIMPROF outputs identified a cluster of tropical affinity that included TDF and GFTr, which had the lowest taxonomic dissimilarity value (Γ^+^ = 9.4%), and separated at 21.5% from the remaining vegetation types (Fig 3A).

For amphibians, vegetation types presented Γ^+^ values lower than 39%, and TDF and MF showed the lowest taxonomic dissimilarity (Γ^+^ = 15.6%). However, no clustering was identified between the different vegetation types (Fig 3B). Reptiles had the highest Γ^+^ dissimilarity between vegetation types. Two groups were identified: a temperate affinity group composed of MF and OF (Γ^+^ = 13%) and a tropical affinity group constituted by GFTr and TDF (Γ^+^ = 13.7%). Additionally, GFTe (Γ^+^ = 41.3%) was identified as an isolated entity (Fig 3C). Similarly, the birds formed two clusters, one with GFTr and TDF and the lowest dissimilarity (Γ^+^ = 5.9%), and another with GFTe and OF (Γ^+^ = 13.4%), while MF was an isolated entity (Γ^+^ = 20.6%) (Fig 3D). In mammals, the analysis identified a group with tropical and temperate vegetation types, including OF and TDF (Γ^+^ = 6.73%); another cluster included MF and GFTe (Γ^+^ = 7.8%); and finally, GFTr was an isolated entity (Γ^+^ = 15.4%) (Fig 3E).

**Fig 4:**
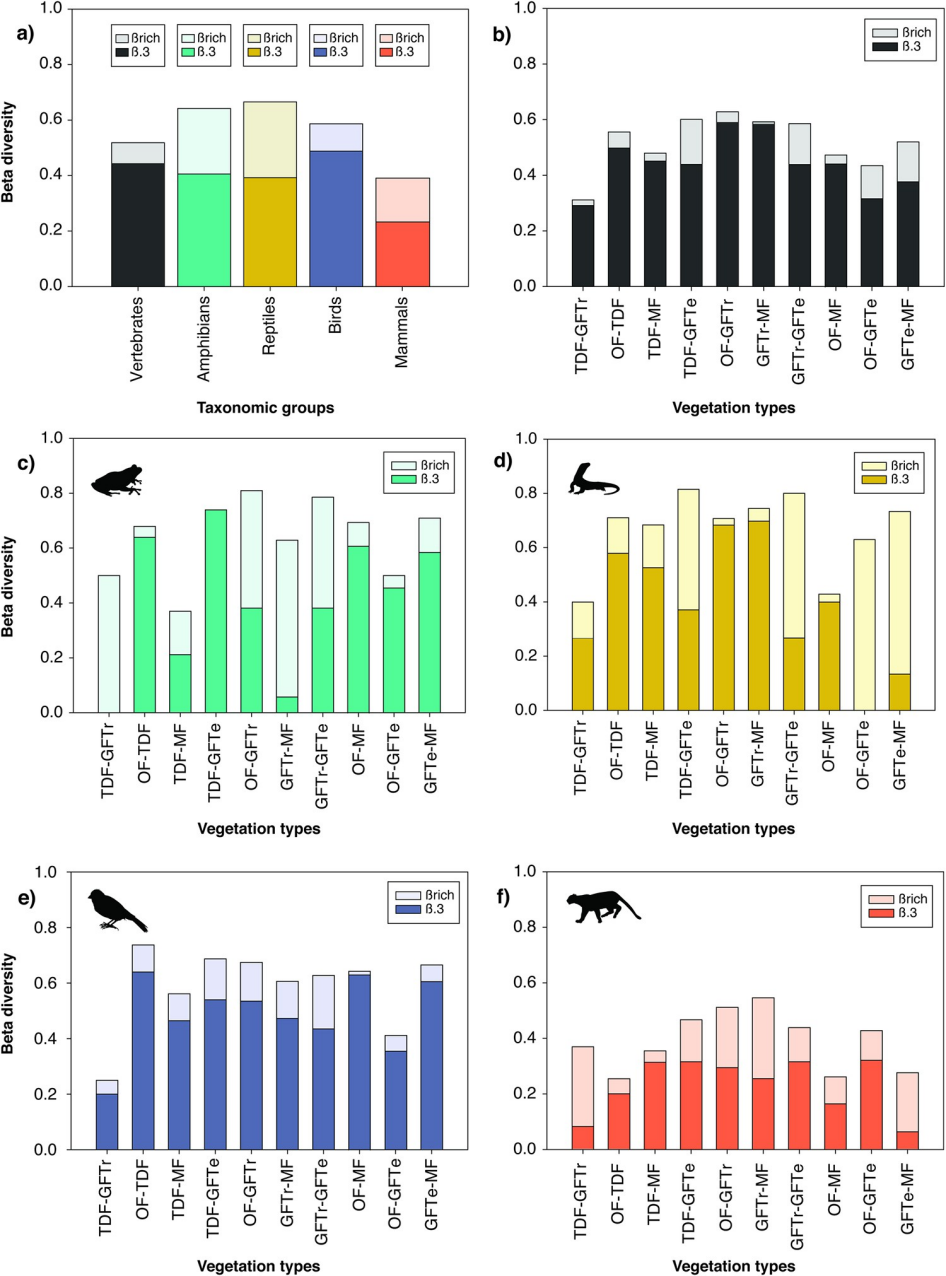
Taxonomic beta diversity of terrestrial vertebrates: Disaggregation on the turnover (*β*_.*3*_) and differences in richness components (*β*_*rich*_) among vegetation types. Total beta diversity by vertebrate group (a); paired beta diversity of vertebrates (b); paired beta diversity of amphibians (c); paired beta diversity of reptiles (d); paired beta diversity of birds (e); and paired beta diversity of mammals (f). Codes: TDF, tropical dry forest; GFTr, tropical gallery forest; OF, oak forest; MF, mixed forest; GFTe, temperate gallery forest.

High overall values were identified for the taxonomic beta diversity of terrestrial vertebrates (*β* = 0.52). The turnover component (*β*_.*3*_) had the highest contribution to taxonomic dissimilarity in the overall comparison and by the biological group (Fig 4; S5 Table). Reptile and mammal groups had the highest and lowest total taxonomic dissimilarity, respectively (*β* = 0.66 and 0.39; Fig 4A). Moreover, in general, paired comparisons showed higher taxonomic dissimilarity, especially between contrasting vegetation types such as OF and GFTr (*β* = 0.589), and lower dissimilarity between the tropical vegetation types TDF and GFTr (*β* = 0.291) (Fig 4B; S5 Table).

For amphibians, a higher contribution of the differences in richness (*β*_*rich*_) component was observed compared with other groups, where beta diversity was generally high (0.50–0.81) in most of the comparisons, except for TDF and MF (*β* = 0.368) (Fig 4C). Similarly, reptiles presented the highest dissimilarity for all comparisons (0.63–0.81), except for the temperate comparison between MF and OF (*β* = 0.43), as well as the tropical TDF and GFTr (*β* = 0.40) (Fig 4D). In birds, differentiation was similar to overall analyses, being high for most comparisons (0.56–0.74), except for the tropical comparison among TDF and GFTr (*β* = 0.25) with the lowest value, and temperate of GFTe and OF (*β* = 0.41) (Fig 4E). In contrast, for mammals, beta diversity was low (0.25–0.47), and the comparisons with the highest differentiation (greater than 0.5) were between tropical and temperate vegetation types GFTr and MF (*β* = 0.55), and OF and GFTr (*β* = 0.51) (Fig 4F; S5 Table).

## 4 Discussion

Taxonomic diversity is a sensitive measure of environmental heterogeneity, unequivocally unveiling spatial patterns of vertebrates within various vegetation types [10,20,60]. Our results showed that tropical areas (TDF and GFTr) form a complex mosaic with the highest taxonomic diversity of terrestrial vertebrates in our study area. The environmental conditions of the tropical regions that allow for resource availability, coupled with higher heterogeneity, could promote a limiting similarity process, which, although not directly linked to their degree of taxonomic relatedness, could explain a higher local diversity and taxonomic complexity than in temperate environments [20]. Therefore, tropical forests are unique and harbor many species and taxonomic levels, some exclusive to these environments (S3 Table). For this reason, tropical forests of the SQPA constitute priority ecosystems for conservation [12,34,61].

### 4.1 Alpha taxonomic diversity

Gallery forests presented extreme values between contrasting environments; the tropical area (GFTr) had the highest species richness and Δ^+^ values, probably because these areas contain resources for a broad array of species, especially during the dry season [62,63]. Conversely, temperate areas present a lower fluctuation of environmental humidity, which could promote a higher independence of the species on water bodies [41]. In this regard, our results suggest that the influence of environmental conditions (temperate *vs*. tropical) could be more critical for explaining the spatial patterns of vertebrate taxonomic structure than the presence of streams (gallery forests).

The values estimated by the model of the Δ^+^ of terrestrial vertebrates indicate that the average change occurs at the class level because the inclusion of species from different taxonomic groups separated at this taxonomic level. In contrast, when the taxonomic groups were examined separately, it showed that the Δ^+^ occurred between the family and order levels, except for reptiles. This could be due to their monophyletic nature since all species considered in this study belong to the order Squamata, promoting an average taxonomic distinctiveness within the family level [64]. These results suggest that reptiles are a group consisting of taxa that can colonize different ecosystems [62,64].

For amphibians, despite the high species richness in the GFTr, its Δ^+^ was higher than expected by the confidence limits (95%), indicating that the taxonomic structure of this group behaves as a random selection from the regional species pool [20]. This result could be explained by the strong seasonality of dry forests that restricts the presence of amphibians to a few months [35,62,63]. In contrast, despite the low species richness in GFTe, the higher number of taxonomic levels promotes a Δ^+^ at the order level due to the inclusion of the order Caudata, which adds two additional species (*Isthmura belli* and *Ambystoma amblycephalum*).

Birds should be considered a priority group because they influence overall patterns of Δ^+^ and their high number of species. The highest richness and Δ^+^ of birds were obtained in tropical areas, which are crucial for species’ development, feeding, and reproduction [14]. Several taxonomic levels were also exclusive to tropical regions, such as the genera *Calocitta*, *Caracara*, *Forpus*, *Geococcyx*, *Heliomaster*, *Polioptila*, *Stelgidopterix*, *Pheugopedius*, and *Thryophilus*, the families Psittacidae, Polioptilidae, and Hirundidae, and the order Psittaciformes. Moreover, GFTe presented Δ^+^ values below the probability funnel because it has the lowest number of orders, families, genera, and species of all vegetation types. These results could be explained by the low resource availability, which could promote the coexistence of phylogenetically close species (between genus and family levels) compared to tropical areas (between family and order levels) [65]. However, compared to other temperate vegetation types (OF and MF), the vegetation structure in GFTe (more closed) could prevent members of the orders Falconiformes and Trogoniformes from frequenting this vegetation type (or being detected).

In contrast, the highest species richness and Δ^+^ of mammals were found in temperate vegetation types, with some species exclusive to these areas (20) (e.g., orders Chiroptera and Rodentia), which contribute notably to taxonomic alpha diversity [66]. Conversely, many species from the order Carnivora were present in all vegetation types (14), possibly due to their high physiological tolerances and generally broader territorial ranges [45,67,68]. Within the SQPA, temperate areas experience less anthropogenic influence as they are located in the higher parts of the study area’s center. Thus, temperate vegetation types could serve as mammal refuges by maintaining the lowest anthropogenic impact within the study area, partially explaining the observed patterns.

### 4.2 Beta taxonomic diversity

The overall patterns of taxonomic dissimilarity showed a strong relatedness among tropical vegetation types (TDF and GFTr), while temperate vegetation types (MF, OF, and GFTe) remained ungrouped. These results suggest that tropical vegetation types have a similar taxonomic structure and exhibit higher taxonomic distinctiveness. In contrast, there was less taxonomic relatedness among the taxonomic levels of temperate vegetation types (Fig 3A). On the other hand, the whole area behaves as the same homogeneous group (TDF, GFTr, OF, MF and GFTe) for amphibian taxonomic distinctiveness (Fig 3B). Similarly, the reptile and bird groups maintained a tropical (TDF and GFTr) and temperate (MF and OF; GFTe and OF, respectively) taxonomic structure (Fig 3C and 3D). In contrast, mammals showed a mixed group (OF and TDF) and a temperate group (MF and GFTe) of vegetation types (Fig 3E). This pattern suggests that amphibian and mammal taxonomic structure may not be particularly sensitive to local environmental conditions in the study area [69].

Beta diversity is scale-dependent since taxa tend to occupy more area units at small scales, decreasing beta diversity [27,70,71]. However, small scales (below 2,500 km^2^) allow us to understand the effect of internal ecological dynamics and environmental conditions on terrestrial vertebrates [15,27]. Despite the local scale effect, beta diversity showed high overall values, supporting the idea that the Mexican Transition Zone is one of the most diverse regions in Mexico regarding vertebrate endemism [12,38]. For example, elevation highly contributes to the *β*_.*3*_ component of vertebrates [67,68]. In the SQPA, the *β*_.*3*_ component contributed more to total beta diversity, probably due to the arrangement of tropical forests in contact with temperate forests, high rate of endemism, and reduced species ranges [27]. In contrast, the *β*_*rich*_ component could be more critical for amphibians and reptiles because of their sensitivity to local environmental conditions (i.e., precipitation and temperature). These patterns might result from historical processes: differences in richness are related to species’ physiological limits, while turnover is more associated with speciation processes, particularly for low-mobility groups [69].

Environmental heterogeneity is a key driver of beta diversity [67]. Although species richness *per se* is not related to beta diversity, when considering species with low dispersal capacity, we found low alpha diversity and, in turn, high beta diversity values, and *vice versa* [15]. In the SQPA, the highest beta diversity was observed in reptiles, followed by amphibians, birds, and mammals. This variation reflects differences in space use, physiological constraints, and dispersal capacity of each taxonomic group [15,69,72]. Thus, high beta diversity in amphibians and reptiles could be related to low dispersal and reduced distribution ranges due to their sensitivity to local environmental conditions [32,70,73]. Previous studies have shown lower bird beta diversity [15,70,74]; however, the SQPA had the lowest mammal beta diversity. This could be related to greater niche breadth, wide mobility, and physiological tolerances that allow mammals to inhabit several environments [15,68,75]. In any case, species turnover is the main component contributing to differentiation when assessing beta diversity patterns in terrestrial vertebrates [10,15,76,77]. Despite the local scale, the SQPA presented high beta diversity (0.39–0.66), reflecting historical ecological processes in taxonomic composition derived from contrasting environments and the limitations imposed on the species (S5 Table) [27,78].

### 4.3 Management and conservation implications

A critical component of conservation is prioritizing areas of ecological importance regarding species richness, threatened species, rarity, and endemism [3,73]. In the SQPA, the species richness of terrestrial vertebrates represents 34.6% of the amphibian species, 14.0% of reptiles, 19.4% of birds, and 42.8% of mammals recorded for Jalisco state in west-central Mexico [79–81]. A significant proportion of species (28.3%) documented in this inventory are endemic to Mexico, most of which were amphibians (72%). However, most of the endemic species lack national (25.4%, Mexican Law) and international (9.5% IUCN; 3.2% CITES) protection (Table 2). These results highlight the need to review the conservation status of vertebrates, especially those with restricted distributions [34,73].

**Table 2:** Conservation status of terrestrial vertebrates of the SQPA.

Taxa	Endemics	NOM-059	IUCN	CITES
Amphibians	13 (72%)	8 (44.4%)	1 (5.5%)	1 (5.5%)
Reptiles	17 (70.8%)	9 (37.5%)	2 (8.3%)	1 (4.2%)
Birds	20 (18.5%)	9 (8.3%)	3 (2.8%)	16 (14.8%)
Mammals	13 (18%)	5 (6.9%)	5 (6.9%)	8 (11.1%)
TDF	27	12	1	18
GFTr	37	19	3	21
OF	22	13	4	10
MF	29	14	4	12
GFTe	18	15	3	8

Conservation status and endemism of vertebrate species of the SQPA by vegetation type. Codes: TDF, tropical dry forest; GFTr, tropical gallery forest; OF, oak forest; MF, mixed forest; GFTe, temperate gallery forest.

Gallery forests are crucial in terrestrial ecosystems as potential wildlife corridors and vital water sources for many species [34,41,62,63]. Despite its limited size, the GFTr boasted the highest species richness and endemic species (122 and 41, respectively) and exhibited the highest beta diversity among the studied vegetation types. These results suggest that the milder seasonality, higher productivity, and complex structure allow more species and taxonomic levels to inhabit this GFTr, even compared to TDF, particularly during the dry season [34]. Additionally, TDFs are often considered to harbor not only high species diversity but also high rates of endemism due to marked seasonal changes, which could impose biotic and abiotic filters on their species (e.g., leaf loss, extreme drought) (S3 Table) [30,34,35,82]. On the other hand, permanent streams in GFTr could buffer abrupt environmental changes imposed on vertebrates by promoting greater niche complementarity [83]. Unfortunately, tropical areas have become the most vulnerable environment of the SQPA because they have the most significant human exposure and one of the highest deforestation rates [12,71,84].

Assessing taxonomic diversity is vital for managing natural protected areas as it enables the recognition of spatially integrated taxon assemblages that characterize geographic areas as a first step to direct local interventions [60,73,85]. However, management strategies should be based on comprehensive *in situ* diagnostics and be representative of biological diversity [23,37,73]. Our results using a multi-species approach provide a foundation for future research to conserve the biodiversity of interconnected tropical and temperate forests [61].

## Supporting information

S1 Table List and conservation status of the vertebrate species of the SQPA. Codes: E (endemic species to Mexico); NOM (Mexican law); IUCN (Red List); CITES. Codes: TDF, tropical dry forest; GFTr, tropical gallery forest; OF, oak forest; MF, mixed forest; GFTe, temperate gallery forest.
(DOCX)

S2 Table Taxonomic aggregation matrix describing the taxonomic levels of the vertebrate species of the SQPA. (DOCX)

S3 Table Exclusive richness in tropical and temperate environments and taxa present in all environments from the SQPA. (DOCX)

S4 Table Results of the average taxonomic distinctiveness analyses overall and at level of taxonomic groups in the SQPA. Codes: TDF, tropical dry forest; GFTr, tropical gallery forest; OF, oak forest; MF, mixed forest; GFTe, temperate gallery forest. Bold numbers represent significant differences (p ≤ 0.05) based on 10,000 permutations.
(DOCX)

S5 Table Taxonomic beta diversity additive partitioning results of terrestrial vertebrates between vegetation types. Codes: *β*_.*3*_, turnover component; *β*_*rich*_, differences in richness component; TDF, tropical dry forest; GFTr tropical gallery forest; OF, oak forest; MF, mixed forest; GFTe, temperate gallery forest. Bold numbers represent values greater than 0.5 of taxonomic dissimilarity.
(DOCX)
