# Supplementary material for: Taxonomic diversity of terrestrial vertebrates in west-central Mexico: Conservation from a multi-taxa perspective
Source: PLoS One. 2024 Oct 9;19(10):e0311770. doi: 10.1371/journal.pone.0311770 (PMC11463785; doi:10.1371/journal.pone.0311770)
Supplement: S1 Table — Codes: E (endemic species to Mexico); NOM (Mexican law); IUCN (Red List); CITES. Codes: TDF, tropical dry forest; GFTr, tropical gallery forest; OF, oak forest; MF, mixed forest; GFTe, temperate gallery forest. (DOCX) [file pone.0311770.s001.docx]

Supplementary material

Taxonomic diversity of terrestrial vertebrates in west-central Mexico: conservation from a multi-taxa perspective

Eliza Álvarez-Grzybowska^1,2^, Verónica Carolina Rosas-Espinoza^2^, Karen Elizabeth Peña-Joya^3^, Ana Luisa Santiago-Pérez^4^, Luis Ignacio Íñiguez-Dávalos^5^, Miguel Ángel Macías-Rodríguez^6^, Fabián Alejandro Rodríguez-Zaragoza^2*^

^1^ Doctorado en Biosistemática, Ecología y Manejo de Recursos Naturales y Agrícolas (BEMARENA), Centro Universitario de Ciencias Biológicas y Agropecuarias, Universidad de Guadalajara, Zapopan, Jalisco, México

^2^ Laboratorio de Ecología Molecular, Microbiología y Taxonomía (LEMITAX), Departamento de Ecología Aplicada, Centro Universitario de Ciencias Biológicas y Agropecuarias, Universidad de Guadalajara, Zaopan, Jalisco, México

^3^ Laboratorio de Ecología, Paisaje y Sociedad, Centro Universitario de la Costa, Universidad de Guadalajara, Puerto Vallarta, Jalisco, México

^4^ Departamento de Producción Forestal, Centro Universitario de Ciencias Biológicas y Agropecuarias, Universidad de Guadalajara, Zapopan, Jalisco, México

^5^ Departamento de Ecología y Recursos Naturales, Centro Universitario de la Costa Sur, Universidad de Guadalajara, Autlán de Navarro, Jalisco, México

^6^ Departamento de Ciencias Ambientales, Centro Universitario de Ciencias Biológicas y Agropecuarias, Universidad de Guadalajara, Zapopan 45200, Jalisco, México

*Corresponding author

E-mail: [fabian.rzaragoza@academicos.udg.mx](mailto:fabian.rzaragoza@academicos.udg.mx) (FARZ)

**Table S1.** **List and conservation status of the vertebrate species of the SQPA.** Codes: E (endemic species to Mexico); NOM (Mexican law); IUCN (Red List); CITES. Codes: TDF, tropical dry forest; GFTr, tropical gallery forest; OF, oak forest; MF, mixed forest; GFTe, temperate gallery forest.

| **Scientific name** | **E** | **NOM** | **IUCN** | **CITES** | **TDF** | **GFTr** | **OF** | **MF** | **GFTe** |
| --- | --- | --- | --- | --- | --- | --- | --- | --- | --- |
| *Agalychnis dacnicolor* | x | - | LC | II | 0 | 1 | 0 | 0 | 0 |
| *Ambystoma amblycephalum* | x | Pr | CR | - | 0 | 0 | 1 | 0 | 1 |
| *Craugastor augusti* | - | - | LC | - | 0 | 0 | 1 | 1 | 0 |
| *Craugastor hobartsmithi* | x | - | LC | - | 0 | 0 | 0 | 0 | 1 |
| *Craugastor occidentalis* | x | - | LC | - | 1 | 1 | 1 | 0 | 0 |
| *Dryophytes arenicolor* | - | - | LC | - | 1 | 1 | 1 | 0 | 0 |
| *Dryophytes eximius* | x | Pr | LC | - | 0 | 1 | 0 | 1 | 0 |
| *Eleutherodactylus nitidus* | x | Pr | LC | - | 1 | 1 | 0 | 1 | 1 |
| *Exerodonta smaragdina* | x | Pr | LC | - | 0 | 1 | 0 | 0 | 1 |
| *Hypopachus variolosus* | - | - | LC | - | 1 | 1 | 0 | 0 | 0 |
| *Incilius marmoreus* | x | - | LC | - | 0 | 1 | 0 | 0 | 0 |
| *Incilius occidentalis* | x | - | LC | - | 1 | 1 | 0 | 1 | 0 |
| *Isthmura bellii* | x | A | LC | - | 0 | 0 | 1 | 0 | 1 |
| *Rana neovolcanica* | x | A | LC | - | 0 | 1 | 0 | 0 | 0 |
| *Rana psilonota* | x | - | LC | - | 0 | 1 | 0 | 0 | 0 |
| *Rhinella horribilis* | - | - | 0 | - | 0 | 1 | 0 | 0 | 0 |
| *Smilisca baudinii* | - | Pr | LC | - | 0 | 1 | 0 | 0 | 0 |
| *Tlalocohyla smithii* | x | Pr | LC | - | 0 | 1 | 0 | 0 | 0 |
| *Aspidoscelis costatus* | x | Pr | LC | - | 0 | 1 | 0 | 0 | 0 |
| *Aspidoscelis gularis* | - | - | LC | - | 1 | 1 | 0 | 0 | 0 |
| *Conopsis nasus* | x | - | LC | - | 0 | 0 | 0 | 1 | 0 |
| *Crotalus triseriatus* | x | - | LC | - | 0 | 0 | 1 | 1 | 0 |
| *Ctenosaura pectinata* | x | A | LC | II | 1 | 1 | 0 | 0 | 0 |
| *Elgaria kingii* | - | Pr | LC | - | 1 | 0 | 0 | 1 | 0 |
| *Geophis bicolor* | x | Pr | DD | - | 0 | 0 | 0 | 1 | 0 |
| *Hypsiglena torquata* | x | Pr | LC | - | 0 | 1 | 0 | 0 | 0 |
| *Leptophis diplotropis* | x | A | LC | - | 0 | 0 | 1 | 0 | 0 |
| *Anolis nebulosus* | x | - | LC | - | 1 | 1 | 1 | 1 | 1 |
| *Plestiodon callicephalus* | - | - | LC | - | 1 | 1 | 1 | 0 | 0 |
| *Plestiodon dugesii* | x | Pr | VU | - | 0 | 0 | 1 | 1 | 0 |
| *Plestiodon lynxe* | x | Pr | LC | - | 0 | 0 | 1 | 1 | 0 |
| *Sceloporus horridus* | x | - | LC | - | 1 | 1 | 0 | 0 | 0 |
| *Sceloporus jarrovii* | - | - | LC | - | 0 | 0 | 0 | 1 | 0 |
| *Sceloporus melanorhinus* | - | - | LC | - | 0 | 0 | 1 | 0 | 0 |
| *Sceloporus scalaris* | x | - | LC | - | 1 | 0 | 1 | 0 | 0 |
| *Sceloporus spinosus* | x | - | LC | - | 0 | 1 | 0 | 0 | 0 |
| *Sceloporus torquatus* | x | - | LC | - | 0 | 1 | 1 | 1 | 0 |
| *Sceloporus utiformis* | x | - | LC | - | 1 | 1 | 0 | 0 | 0 |
| *Senticolis triaspis* | - | - | LC | - | 0 | 1 | 0 | 0 | 0 |
| *Storeria storerioides* | x | - | LC | - | 0 | 0 | 1 | 1 | 1 |
| *Tantilla bocourti* | x | - | LC | - | 0 | 0 | 1 | 1 | 0 |
| *Thamnophis cyrtopsis* | - | A | LC | - | 0 | 0 | 1 | 0 | 1 |
| *Accipiter striatus* | - | Pr | LC | - | 1 | 1 | 1 | 1 | 1 |
| *Peucaea humeralis* | x | - | LC | - | 1 | 1 | 0 | 1 | 0 |
| *Saucerottia beryllina* | - | - | LC | II | 1 | 1 | 1 | 0 | 1 |
| *Ramosomyia violiceps* | - | - | LC | II | 1 | 1 | 0 | 0 | 0 |
| *Aphelocoma ultramarina* | x | - | LC | - | 0 | 0 | 1 | 0 | 1 |
| *Attila spadiceus* | - | - | LC | - | 1 | 0 | 0 | 1 | 0 |
| *Baeolophus wollweberi* | - | - | LC | - | 0 | 0 | 1 | 1 | 1 |
| *Buteo jamaicensis* | - | - | LC | - | 1 | 1 | 1 | 0 | 1 |
| *Buteo nitidus* | - | - | LC | - | 1 | 1 | 0 | 1 | 0 |
| *Buteogallus anthracinus* | - | Pr | LC | II | 0 | 1 | 0 | 0 | 1 |
| *Calocitta colliei* | x | - | 0 | - | 1 | 1 | 0 | 0 | 0 |
| *Calocitta formosa* | - | - | 0 | - | 1 | 1 | 0 | 0 | 0 |
| *Caracara plancus* | - | - | LC | II | 1 | 1 | 0 | 0 | 0 |
| *Cardellina pusilla* | - | - | LC | - | 0 | 1 | 1 | 0 | 1 |
| *Haemorhous mexicanus* | - | - | LC | - | 1 | 1 | 0 | 1 | 0 |
| *Cardellina rubrifrons* | - | - | LC | - | 0 | 0 | 1 | 0 | 1 |
| *Spinus notatus* | - | - | LC | - | 0 | 1 | 0 | 1 | 1 |
| *Cathartes aura* | - | - | LC | - | 1 | 1 | 1 | 1 | 1 |
| *Catharus aurantiirostris* | - | - | LC | - | 1 | 1 | 0 | 1 | 1 |
| *Catharus guttatus* | - | - | LC | - | 0 | 0 | 1 | 0 | 1 |
| *Certhia americana* | - | - | LC | - | 0 | 0 | 1 | 1 | 1 |
| *Colinus virginianus* | - | - | NT | - | 0 | 0 | 0 | 1 | 0 |
| *Contopus pertinax* | - | - | LC | - | 0 | 1 | 1 | 1 | 1 |
| *Coragyps atratus* | - | - | LC | - | 1 | 1 | 1 | 1 | 1 |
| *Corvus corax* | - | - | LC | - | 1 | 1 | 1 | 1 | 1 |
| *Cynanthus latirostris* | - | - | LC | II | 1 | 1 | 1 | 1 | 0 |
| *Cyrtonyx montezumae* | - | Pr | LC | - | 0 | 0 | 1 | 0 | 0 |
| *Empidonax fulvifrons* | - | - | LC | - | 0 | 0 | 1 | 1 | 1 |
| *Empidonax minimus* | - | - | LC | - | 0 | 1 | 0 | 0 | 0 |
| *Empidonax occidentalis* | - | - | LC | - | 0 | 0 | 1 | 1 | 1 |
| *Cardellina rubra* | x | - | LC | - | 0 | 0 | 0 | 1 | 0 |
| *Eugenes fulgens* | - | - | LC | II | 0 | 0 | 0 | 1 | 0 |
| *Chlorophonia elegantissima* | - | - | LC | - | 1 | 1 | 0 | 1 | 0 |
| *Falco peregrinus* | - | Pr | LC | I | 0 | 1 | 1 | 0 | 0 |
| *Falco sparverius* | - | - | LC | II | 1 | 1 | 0 | 0 | 0 |
| *Forpus cyanopygius* | x | Pr | NT | II | 1 | 1 | 0 | 0 | 0 |
| *Geococcyx velox* | - | - | LC | - | 1 | 1 | 0 | 0 | 0 |
| *Heliomaster constantii* | - | - | LC | II | 1 | 1 | 0 | 0 | 0 |
| *Basilinna leucotis* | - | - | LC | II | 0 | 0 | 1 | 0 | 1 |
| *Icterus abeillei* | x | - | LC | - | 0 | 0 | 0 | 1 | 0 |
| *Icterus bullockii* | - | - | 0 | - | 1 | 0 | 0 | 1 | 0 |
| *Icterus cucullatus* | - | - | LC | - | 1 | 1 | 0 | 1 | 0 |
| *Icterus parisorum* | - | - | LC | - | 1 | 0 | 0 | 0 | 0 |
| *Icterus wagleri* | - | - | LC | - | 1 | 1 | 0 | 1 | 0 |
| *Lepidocolaptes leucogaster* | x | - | LC | - | 1 | 1 | 1 | 0 | 1 |
| *Leptotila verreauxi* | - | - | LC | - | 1 | 1 | 0 | 1 | 0 |
| *Melanerpes formicivorus* | - | - | LC | - | 0 | 0 | 1 | 0 | 0 |
| *Melanerpes chrysogenys* | x | - | LC | - | 1 | 1 | 0 | 0 | 0 |
| *Melanotis caerulescens* | x | - | LC | - | 1 | 1 | 0 | 0 | 1 |
| *Melozone kieneri* | x | - | LC | - | 1 | 1 | 0 | 1 | 0 |
| *Mitrephanes phaeocercus* | - | - | LC | - | 0 | 1 | 1 | 0 | 1 |
| *Mniotilta varia* | - | - | LC | - | 1 | 1 | 0 | 1 | 1 |
| *Molothrus aeneus* | - | - | LC | - | 1 | 0 | 0 | 1 | 0 |
| *Myadestes occidentalis* | - | Pr | LC | - | 0 | 0 | 1 | 0 | 1 |
| *Myiarchus cinerascens* | - | - | LC | - | 1 | 1 | 0 | 1 | 1 |
| *Myiarchus tuberculifer* | - | - | LC | - | 1 | 0 | 0 | 0 | 1 |
| *Myioborus miniatus* | - | - | LC | - | 0 | 0 | 1 | 1 | 1 |
| *Myioborus pictus* | - | - | LC | - | 1 | 0 | 1 | 1 | 1 |
| *Myiopagis viridicata* | - | - | LC | - | 1 | 0 | 0 | 1 | 0 |
| *Leiothlypis celata* | - | - | LC | - | 1 | 1 | 1 | 0 | 1 |
| *Ortalis poliocephala* | x | - | LC | - | 1 | 1 | 0 | 1 | 0 |
| *Pachyramphus aglaiae* | - | - | LC | - | 1 | 1 | 1 | 0 | 1 |
| *Parabuteo unicinctus* | - | Pr | LC | II | 1 | 1 | 0 | 1 | 0 |
| *Oreothlypis superciliosa* | - | - | LC | - | 0 | 1 | 1 | 0 | 1 |
| *Passerina leclancherii* | x | - | LC | - | 1 | 1 | 0 | 1 | 0 |
| *Patagioenas fasciata* | - | - | LC | - | 0 | 0 | 1 | 0 | 1 |
| *Peucaea ruficauda* | - | - | LC | - | 1 | 1 | 0 | 1 | 0 |
| *Peucedramus taeniatus* | - | - | LC | - | 0 | 0 | 1 | 0 | 0 |
| *Pheucticus melanocephalus* | - | - | LC | - | 1 | 1 | 1 | 0 | 1 |
| *Piaya cayana* | - | - | LC | - | 1 | 1 | 0 | 1 | 1 |
| *Dryobates arizonae* | - | - | 0 | - | 0 | 0 | 1 | 0 | 0 |
| *Dryobates villosus* | - | - | 0 | - | 0 | 0 | 1 | 1 | 0 |
| *Pipilo ocai* | x | - | LC | - | 0 | 1 | 0 | 0 | 0 |
| *Piranga bidentata* | - | - | LC | - | 1 | 1 | 1 | 1 | 0 |
| *Piranga erythrocephala* | x | - | LC | - | 0 | 0 | 1 | 1 | 1 |
| *Piranga flava* | - | - | 0 | - | 0 | 0 | 1 | 0 | 1 |
| *Piranga ludoviciana* | - | - | LC | - | 0 | 0 | 1 | 1 | 0 |
| *Polioptila caerulea* | - | - | LC | - | 1 | 1 | 0 | 0 | 0 |
| *Polioptila nigriceps* | x | - | LC | - | 1 | 1 | 0 | 0 | 0 |
| *Ridgwayia pinicola* | x | Pr | LC | - | 0 | 0 | 0 | 0 | 1 |
| *Corthylio calendula* | - | - | LC | - | 0 | 0 | 1 | 0 | 1 |
| *Parkesia motacilla* | - | - | LC | - | 0 | 0 | 0 | 1 | 1 |
| *Selasphorus rufus* | - | - | NT | II | 0 | 1 | 1 | 0 | 0 |
| *Selasphorus sasin* | - | - | LC | II | 0 | 0 | 1 | 1 | 0 |
| *Selasphorus platycercus* | - | - | LC | II | 0 | 0 | 0 | 1 | 0 |
| *Setophaga coronata* | - | - | LC | - | 1 | 1 | 1 | 1 | 1 |
| *Setophaga graciae* | - | - | LC | - | 0 | 0 | 1 | 1 | 0 |
| *Setophaga nigrescens* | - | - | LC | - | 1 | 1 | 1 | 1 | 1 |
| *Setophaga occidentalis* | - | - | LC | - | 0 | 0 | 1 | 1 | 1 |
| *Setophaga townsendi* | - | - | LC | - | 1 | 0 | 1 | 1 | 1 |
| *Sialia sialis* | - | - | LC | - | 0 | 0 | 1 | 1 | 0 |
| *Spinus psaltria* | - | - | LC | - | 1 | 1 | 0 | 0 | 0 |
| *Sphyrapicus varius* | - | - | LC | - | 0 | 0 | 1 | 0 | 0 |
| *Stelgidopteryx serripennis* | - | - | LC | - | 1 | 1 | 0 | 0 | 0 |
| *Tilmatura dupontii* | - | A | LC | II | 1 | 1 | 0 | 1 | 0 |
| *Pheugopedius felix* | x | - | LC | - | 1 | 1 | 0 | 0 | 0 |
| *Thryophilus sinaloa* | x | - | LC | - | 1 | 1 | 0 | 0 | 0 |
| *Troglodytes aedon* | - | - | LC | - | 1 | 1 | 1 | 0 | 1 |
| *Trogon elegans* | - | - | 0 | - | 0 | 0 | 1 | 1 | 0 |
| *Turdus assimilis* | - | - | LC | - | 0 | 0 | 1 | 1 | 1 |
| *Turdus migratorius* | - | - | LC | - | 0 | 1 | 1 | 1 | 1 |
| *Turdus rufopalliatus* | x | - | LC | - | 0 | 1 | 0 | 0 | 1 |
| *Tyrannus verticalis* | - | - | LC | - | 1 | 1 | 0 | 0 | 0 |
| *Vireo cassini* | - | - | LC | - | 0 | 0 | 1 | 0 | 1 |
| *Vireo flavoviridis* | - | - | LC | - | 0 | 0 | 0 | 0 | 1 |
| *Vireo hypochryseus* | x | - | 0 | - | 1 | 1 | 0 | 0 | 0 |
| *Zenaida asiatica* | - | - | LC | - | 1 | 0 | 0 | 0 | 1 |
| *Zenaida macroura* | - | - | LC | - | 0 | 0 | 1 | 0 | 0 |
| *Didelphis virginiana* | - | - | LC | - | 1 | 1 | 1 | 1 | 1 |
| *Sylvilagus cunicularius* | x | - | LC | - | 0 | 0 | 0 | 1 | 0 |
| *Sylvilagus floridanus* | - | - | LC | - | 1 | 0 | 1 | 1 | 0 |
| *Sciurus aureogaster* | - | - | LC | - | 0 | 0 | 1 | 1 | 1 |
| *Sciurus colliaei* | x | - | LC | - | 1 | 0 | 1 | 1 | 1 |
| *Sciurus nayaritensis* | - | - | LC | - | 0 | 0 | 0 | 1 | 0 |
| *Otospermophilus variegatus* | - | - | LC | - | 1 | 0 | 1 | 1 | 1 |
| *Herpailurus yagouaroundi* | - | A | LC | I | 1 | 1 | 1 | 1 | 1 |
| *Leopardus pardalis* | - | P | LC | I | 1 | 1 | 1 | 0 | 1 |
| *Leopardus wiedii* | - | P | NT | I | 1 | 1 | 0 | 1 | 1 |
| *Lynx rufus* | - | - | LC | II | 1 | 0 | 1 | 1 | 0 |
| *Puma concolor* | - | - | LC | II | 1 | 1 | 1 | 1 | 1 |
| *Panthera onca* | - | P | NT | I | 1 | 1 | 0 | 1 | 1 |
| *Canis latrans* | - | - | LC | - | 1 | 0 | 1 | 1 | 0 |
| *Urocyon cinereoargenteus* | - | - | LC | - | 1 | 0 | 1 | 1 | 0 |
| *Conepatus leuconotus* | - | - | LC | - | 1 | 1 | 1 | 1 | 1 |
| *Mephitis macroura* | - | - | LC | - | 1 | 1 | 0 | 1 | 1 |
| *Spilogale angustifrons* | - | - | LC | - | 1 | 0 | 1 | 1 | 0 |
| *Dasypus novemcinctus* | - | - | LC | - | 1 | 1 | 1 | 1 | 1 |
| *Lontra longicaudis* | - | - | NT | I | 0 | 1 | 0 | 0 | 0 |
| *Mustela frenata* | - | - | LC | - | 1 | 1 | 1 | 1 | 1 |
| *Bassariscus astutus* | - | - | LC | - | 1 | 0 | 1 | 1 | 0 |
| *Nasua narica* | - | - | LC | III | 1 | 1 | 1 | 1 | 1 |
| *Procyon lotor* | - | - | LC | - | 1 | 1 | 1 | 1 | 1 |
| *Dicotyles tajacu* | - | - | LC | - | 1 | 1 | 1 | 1 | 1 |
| *Odocoileus virginianus* | - | - | LC | - | 1 | 1 | 1 | 1 | 1 |
| *Desmodus rotundus* | - | - | LC | - | 1 | 1 | 0 | 0 | 0 |
| *Micronycteris microtis* | - | - | LC | - | 1 | 1 | 0 | 0 | 0 |
| *Anoura geoffroyi* | - | - | LC | - | 1 | 1 | 1 | 1 | 0 |
| *Choeroniscus godmani* | - | - | LC | - | 1 | 1 | 1 | 0 | 0 |
| *Choeroniscus mexicana* | - | - | 0 | - | 1 | 0 | 1 | 0 | 0 |
| *Artibeus jamaicensis* | - | - | LC | - | 1 | 1 | 1 | 0 | 1 |
| *Artibeus lituratus* | - | - | LC | - | 1 | 1 | 1 | 0 | 0 |
| *Chiroderma salvini* | - | - | LC | - | 1 | 1 | 0 | 0 | 0 |
| *Dermanura tolteca* | - | - | LC | - | 1 | 1 | 1 | 1 | 1 |
| *Enchisthenes hartii* | - | Pr | LC | - | 1 | 0 | 0 | 1 | 1 |
| *Sturnira hondurensis* | - | - | LC | - | 1 | 1 | 1 | 1 | 1 |
| *Sturnira parvidens* | - | - | LC | - | 1 | 1 | 1 | 1 | 1 |
| *Leptpnycteris nivalis* | - | A | EN | - | 0 | 0 | 0 | 1 | 1 |
| *Glossophaga sp.* | - | - | - | - | 1 | 1 | 1 | 1 | 1 |
| *Pteronotus parnellii* | - | - | 0 | - | 1 | 1 | 1 | 1 | 1 |
| *Tadarida brasiliensis* | - | - | LC | - | 1 | 0 | 0 | 0 | 0 |
| *Myotis auriculus* | - | - | LC | - | 0 | 0 | 1 | 1 | 0 |
| *Myotis californicus* | - | - | LC | - | 1 | 0 | 1 | 1 | 1 |
| *Myotis fortidens* | - | - | LC | - | 1 | 1 | 0 | 0 | 1 |
| *Myotis thysanodes* | - | - | LC | - | 0 | 0 | 0 | 1 | 1 |
| *Myotis yumanensis* | - | - | LC | - | 1 | 0 | 1 | 1 | 1 |
| *Corynorhinus mexicanus* | x | - | NT | - | 0 | 0 | 1 | 1 | 1 |
| *Eptesicus furinalis* | - | - | LC | - | 1 | 1 | 0 | 0 | 0 |
| *Eptesicus fuscus* | - | - | LC | - | 1 | 1 | 1 | 1 | 1 |
| *Lasiurus frantzii* | - | - | LC | - | 0 | 0 | 1 | 1 | 1 |
| *Lasiurus cinereus* | - | - | LC | - | 0 | 0 | 0 | 1 | 1 |
| *Lasiurus intermedius* | - | - | LC | - | 0 | 0 | 0 | 1 | 1 |
| *Lasiurus xanthinus* | - | - | LC | - | 0 | 1 | 0 | 1 | 1 |
| *Rhogeessa alleni* | x | - | LC | - | 1 | 1 | 0 | 1 | 0 |
| *Rhogeessa gracilis* | x | - | 0 | - | 1 | 1 | 0 | 0 | 0 |
| *Rhogeessa parvula* | x | - | LC | - | 1 | 1 | 1 | 0 | 0 |
| *Pappogeomys bulleri* | x | - | LC | - | 0 | 0 | 1 | 1 | 0 |
| *Heteromys pictus* | - | - | LC | - | 1 | 0 | 1 | 1 | 0 |
| *Neotoma mexicana* | - | - | LC | - | 0 | 0 | 1 | 1 | 1 |
| *Sigmodon alleni* | x | - | VU | - | 1 | 0 | 1 | 1 | 0 |
| *Sigmodon hispidus* | - | - | LC | - | 1 | 0 | 1 | 0 | 0 |
| *Reithrodontomys megalotis* | - | - | LC | - | 0 | 0 | 0 | 1 | 1 |
| *Reithrodontomys zacatecae* | x | - | LC | - | 0 | 0 | 0 | 1 | 1 |
| *Reithrodontomys fulvescens* | - | - | LC | - | 1 | 0 | 1 | 1 | 1 |
| *Baiomys musculus* | x | - | LC | - | 1 | 0 | 1 | 0 | 0 |
| *Peromyscus spicilegus* | x | - | LC | - | 0 | 0 | 0 | 1 | 0 |
| *Peromyscus hylocetes* | x | - | LC | - | 0 | 1 | 0 | 1 | 1 |
| *Peromyscus boylii* | - | - | LC | - | 1 | 0 | 1 | 1 | 1 |
| *Peromyscus maniculatus* | - | - | LC | - | 0 | 0 | 1 | 1 | 1 |
| *Peromyscus melanotis* | x | - | LC | - | 0 | 0 | 1 | 1 | 1 |
| *Cryptotis parvus* | - | - | LC | - | 0 | 0 | 0 | 1 | 1 |
