# Supplementary material for: Taxonomic diversity of terrestrial vertebrates in west-central Mexico: Conservation from a multi-taxa perspective
Source: PLoS One. 2024 Oct 9;19(10):e0311770. doi: 10.1371/journal.pone.0311770 (PMC11463785; doi:10.1371/journal.pone.0311770)
Supplement: S2 Table — (DOCX) [file pone.0311770.s002.docx]

Supplementary material

Taxonomic diversity of terrestrial vertebrates in west-central Mexico: conservation from a multi-taxa perspective

Eliza Álvarez-Grzybowska^1,2^, Verónica Carolina Rosas-Espinoza^2^, Karen Elizabeth Peña-Joya^3^, Ana Luisa Santiago-Pérez^4^, Luis Ignacio Íñiguez-Dávalos^5^, Miguel Ángel Macías-Rodríguez^6^, Fabián Alejandro Rodríguez-Zaragoza^2*^

^1^ Doctorado en Biosistemática, Ecología y Manejo de Recursos Naturales y Agrícolas (BEMARENA), Centro Universitario de Ciencias Biológicas y Agropecuarias, Universidad de Guadalajara, Zapopan, Jalisco, México

^2^ Laboratorio de Ecología Molecular, Microbiología y Taxonomía (LEMITAX), Departamento de Ecología Aplicada, Centro Universitario de Ciencias Biológicas y Agropecuarias, Universidad de Guadalajara, Zaopan, Jalisco, México

^3^ Laboratorio de Ecología, Paisaje y Sociedad, Centro Universitario de la Costa, Universidad de Guadalajara, Puerto Vallarta, Jalisco, México

^4^ Departamento de Producción Forestal, Centro Universitario de Ciencias Biológicas y Agropecuarias, Universidad de Guadalajara, Zapopan, Jalisco, México

^5^ Departamento de Ecología y Recursos Naturales, Centro Universitario de la Costa Sur, Universidad de Guadalajara, Autlán de Navarro, Jalisco, México

^6^ Departamento de Ciencias Ambientales, Centro Universitario de Ciencias Biológicas y Agropecuarias, Universidad de Guadalajara, Zapopan 45200, Jalisco, México

*Corresponding author

E-mail: [fabian.rzaragoza@academicos.udg.mx](mailto:fabian.rzaragoza@academicos.udg.mx) (FARZ)

**Table S2.** **Taxonomic aggregation matrix describing the taxonomic levels of the vertebrate species of the SQPA.**

| **Scientific names** | **Genera** | **Family** | **Order** | **Class** |
| --- | --- | --- | --- | --- |
| *Agalychnis dacnicolor* | Agalychnis | Phyllomedusidae | Anura | Amphibia |
| *Ambystoma amblycephalum* | Ambystoma | Ambystomatidae | Caudata | Amphibia |
| *Craugastor augusti* | Craugastor | Craugastoridae | Anura | Amphibia |
| *Craugastor hobartsmithi* | Craugastor | Craugastoridae | Anura | Amphibia |
| *Craugastor occidentalis* | Craugastor | Craugastoridae | Anura | Amphibia |
| *Dryophytes arenicolor* | Dryophytes | Hylidae | Anura | Amphibia |
| *Dryophytes eximius* | Dryophytes | Hylidae | Anura | Amphibia |
| *Eleutherodactylus nitidus* | Eleutherodactylus | Eleutherodactylidae | Anura | Amphibia |
| *Exerodonta smaragdina* | Exerodonta | Hylidae | Anura | Amphibia |
| *Hypopachus variolosus* | Hypopachus | Microhylidae | Anura | Amphibia |
| *Incilius marmoreus* | Incilius | Bufonidae | Anura | Amphibia |
| *Incilius occidentalis* | Incilius | Bufonidae | Anura | Amphibia |
| *Isthmura bellii* | Isthmura | Plethodontidae | Caudata | Amphibia |
| *Rana neovolcanica* | Rana | Ranidae | Anura | Amphibia |
| *Rana psilonota* | Rana | Ranidae | Anura | Amphibia |
| *Rhinella horribilis* | Rhinella | Bufonidae | Anura | Amphibia |
| *Smilisca baudinii* | Smilisca | Hylidae | Anura | Amphibia |
| *Tlalocohyla smithii* | Tlalocohyla | Hylidae | Anura | Amphibia |
| *Aspidoscelis costatus* | Aspidoscelis | Teiidae | Squamata | Reptilia |
| *Aspidoscelis gularis* | Aspidoscelis | Teiidae | Squamata | Reptilia |
| *Conopsis nasus* | Conopsis | Colubridae | Squamata | Reptilia |
| *Crotalus triseriatus* | Crotalus | Viperidae | Squamata | Reptilia |
| *Ctenosaura pectinata* | Ctenosaura | Iguanidae | Squamata | Reptilia |
| *Elgaria kingii* | Elgaria | Anguidae | Squamata | Reptilia |
| *Geophis bicolor* | Geophis | Colubridae | Squamata | Reptilia |
| *Hypsiglena torquata* | Hypsiglena | Colubridae | Squamata | Reptilia |
| *Leptophis diplotropis* | Leptophis | Colubridae | Squamata | Reptilia |
| *Anolis nebulosus* | Anolis | Datyloidae | Squamata | Reptilia |
| *Plestiodon callicephalus* | Plestiodon | Scincidae | Squamata | Reptilia |
| *Plestiodon dugesii* | Plestiodon | Scincidae | Squamata | Reptilia |
| *Plestiodon lynxe* | Plestiodon | Scincidae | Squamata | Reptilia |
| *Sceloporus horridus* | Sceloporus | Phrynosomatidae | Squamata | Reptilia |
| *Sceloporus jarrovii* | Sceloporus | Phrynosomatidae | Squamata | Reptilia |
| *Sceloporus melanorhinus* | Sceloporus | Phrynosomatidae | Squamata | Reptilia |
| *Sceloporus scalaris* | Sceloporus | Phrynosomatidae | Squamata | Reptilia |
| *Sceloporus spinosus* | Sceloporus | Phrynosomatidae | Squamata | Reptilia |
| *Sceloporus torquatus* | Sceloporus | Phrynosomatidae | Squamata | Reptilia |
| *Sceloporus utiformis* | Sceloporus | Phrynosomatidae | Squamata | Reptilia |
| *Senticolis triaspis* | Senticolis | Colubridae | Squamata | Reptilia |
| *Storeria storerioides* | Storeria | Colubridae | Squamata | Reptilia |
| *Tantilla bocourti* | Tantilla | Colubridae | Squamata | Reptilia |
| *Thamnophis cyrtopsis* | Thamnophis | Colubridae | Squamata | Reptilia |
| *Accipiter striatus* | Accipiter | Accipitridae | Accipitriformes | Aves |
| *Peucaea humeralis* | Peucaea | Passerellidae | Passeriformes | Aves |
| *Saucerottia beryllina* | Saucerottia | Trochilidae | Caprimulgiformes | Aves |
| *Ramosomyia violiceps* | Ramosomyia | Trochilidae | Caprimulgiformes | Aves |
| *Aphelocoma ultramarina* | Aphelocoma | Corvidae | Passeriformes | Aves |
| *Attila spadiceus* | Attila | Tyrannidae | Passeriformes | Aves |
| *Baeolophus wollweberi* | Baeolophus | Paridae | Passeriformes | Aves |
| *Buteo jamaicensis* | Buteo | Accipitridae | Accipitriformes | Aves |
| *Buteo nitidus* | Buteo | Accipitridae | Accipitriformes | Aves |
| *Buteogallus anthracinus* | Buteogallus | Accipitridae | Accipitriformes | Aves |
| *Calocitta colliei* | Calocitta | Corvidae | Passeriformes | Aves |
| *Calocitta formosa* | Calocitta | Corvidae | Passeriformes | Aves |
| *Caracara plancus* | Caracara | Falconidae | Falconiformes | Aves |
| *Cardellina pusilla* | Cardellina | Parulidae | Passeriformes | Aves |
| *Haemorhous mexicanus* | Haemorhous | Fringillidae | Passeriformes | Aves |
| *Cardellina rubrifrons* | Cardellina | Parulidae | Passeriformes | Aves |
| *Spinus notatus* | Spinus | Fringillidae | Passeriformes | Aves |
| *Cathartes aura* | Cathartes | Cathartidae | Cathartiformes | Aves |
| *Catharus aurantiirostris* | Catharus | Turdidae | Passeriformes | Aves |
| *Catharus guttatus* | Catharus | Turdidae | Passeriformes | Aves |
| *Certhia americana* | Certhia | Certhiidae | Passeriformes | Aves |
| *Colinus virginianus* | Colinus | Odontophoridae | Galliformes | Aves |
| *Contopus pertinax* | Contopus | Tyrannidae | Passeriformes | Aves |
| *Coragyps atratus* | Coragyps | Cathartidae | Cathartiformes | Aves |
| *Corvus corax* | Corvus | Corvidae | Passeriformes | Aves |
| *Cynanthus latirostris* | Cynanthus | Trochilidae | Caprimulgiformes | Aves |
| *Cyrtonyx montezumae* | Cyrtonyx | Odontophoridae | Galliformes | Aves |
| *Empidonax fulvifrons* | Empidonax | Tyrannidae | Passeriformes | Aves |
| *Empidonax minimus* | Empidonax | Tyrannidae | Passeriformes | Aves |
| *Empidonax occidentalis* | Empidonax | Tyrannidae | Passeriformes | Aves |
| *Cardellina rubra* | Cardellina | Parulidae | Passeriformes | Aves |
| *Eugenes fulgens* | Eugenes | Trochilidae | Caprimulgiformes | Aves |
| *Chlorophonia elegantissima* | Chlorophonia | Fringillidae | Passeriformes | Aves |
| *Falco peregrinus* | Falco | Falconidae | Falconiformes | Aves |
| *Falco sparverius* | Falco | Falconidae | Falconiformes | Aves |
| *Forpus cyanopygius* | Forpus | Psittacidae | Psittaciformes | Aves |
| *Geococcyx velox* | Geococcyx | Cuculidae | Cuculiformes | Aves |
| *Heliomaster constantii* | Heliomaster | Trochilidae | Caprimulgiformes | Aves |
| *Basilinna leucotis* | Basilinna | Trochilidae | Caprimulgiformes | Aves |
| *Icterus abeillei* | Icterus | Icteridae | Passeriformes | Aves |
| *Icterus bullockii* | Icterus | Icteridae | Passeriformes | Aves |
| *Icterus cucullatus* | Icterus | Icteridae | Passeriformes | Aves |
| *Icterus parisorum* | Icterus | Icteridae | Passeriformes | Aves |
| *Icterus wagleri* | Icterus | Icteridae | Passeriformes | Aves |
| *Lepidocolaptes leucogaster* | Lepidocolaptes | Furnariidae | Passeriformes | Aves |
| *Leptotila verreauxi* | Leptotila | Columbidae | Columbiformes | Aves |
| *Melanerpes formicivorus* | Melanerpes | Picidae | Piciformes | Aves |
| *Melanerpes chrysogenys* | Melanerpes | Picidae | Piciformes | Aves |
| *Melanotis caerulescens* | Melanotis | Mimidae | Passeriformes | Aves |
| *Melozone kieneri* | Melozone | Passerellidae | Passeriformes | Aves |
| *Mitrephanes phaeocercus* | Mitrephanes | Tyrannidae | Passeriformes | Aves |
| *Mniotilta varia* | Mniotilta | Parulidae | Passeriformes | Aves |
| *Molothrus aeneus* | Molothrus | Icteridae | Passeriformes | Aves |
| *Myadestes occidentalis* | Myadestes | Turdidae | Passeriformes | Aves |
| *Myiarchus cinerascens* | Myiarchus | Tyrannidae | Passeriformes | Aves |
| *Myiarchus tuberculifer* | Myiarchus | Tyrannidae | Passeriformes | Aves |
| *Myioborus miniatus* | Myioborus | Parulidae | Passeriformes | Aves |
| *Myioborus pictus* | Myioborus | Parulidae | Passeriformes | Aves |
| *Myiopagis viridicata* | Myiopagis | Tyrannidae | Passeriformes | Aves |
| *Leiothlypis celata* | Leiothlypis | Parulidae | Passeriformes | Aves |
| *Ortalis poliocephala* | Ortalis | Cracidae | Galliformes | Aves |
| *Pachyramphus aglaiae* | Pachyramphus | Tityridae | Passeriformes | Aves |
| *Parabuteo unicinctus* | Parabuteo | Accipitridae | Accipitriformes | Aves |
| *Oreothlypis superciliosa* | Oreothlypis | Parulidae | Passeriformes | Aves |
| *Passerina leclancherii* | Passerina | Cardinalidae | Passeriformes | Aves |
| *Patagioenas fasciata* | Patagioenas | Columbidae | Columbiformes | Aves |
| *Peucaea ruficauda* | Peucaea | Passerellidae | Passeriformes | Aves |
| *Peucedramus taeniatus* | Peucedramus | Peucedramidae | Passeriformes | Aves |
| *Pheucticus melanocephalus* | Pheucticus | Cardinalidae | Passeriformes | Aves |
| *Piaya cayana* | Piaya | Cuculidae | Cuculiformes | Aves |
| *Dryobates arizonae* | Dryobates | Picidae | Piciformes | Aves |
| *Dryobates villosus* | Dryobates | Picidae | Piciformes | Aves |
| *Pipilo ocai* | Pipilo | Passerellidae | Passeriformes | Aves |
| *Piranga bidentata* | Piranga | Cardinalidae | Passeriformes | Aves |
| *Piranga erythrocephala* | Piranga | Cardinalidae | Passeriformes | Aves |
| *Piranga flava* | Piranga | Cardinalidae | Passeriformes | Aves |
| *Piranga ludoviciana* | Piranga | Cardinalidae | Passeriformes | Aves |
| *Polioptila caerulea* | Polioptila | Polioptilidae | Passeriformes | Aves |
| *Polioptila nigriceps* | Polioptila | Polioptilidae | Passeriformes | Aves |
| *Ridgwayia pinicola* | Ridgwayia | Turdidae | Passeriformes | Aves |
| *Corthylio calendula* | Regulus | Regulidae | Passeriformes | Aves |
| *Parkesia motacilla* | Parkesia | Parulidae | Passeriformes | Aves |
| *Selasphorus rufus* | Selasphorus | Trochilidae | Caprimulgiformes | Aves |
| *Selasphorus sasin* | Selasphorus | Trochilidae | Caprimulgiformes | Aves |
| *Selasphorus platycercus* | Selasphorus | Trochilidae | Caprimulgiformes | Aves |
| *Setophaga coronata* | Setophaga | Parulidae | Passeriformes | Aves |
| *Setophaga graciae* | Setophaga | Parulidae | Passeriformes | Aves |
| *Setophaga nigrescens* | Setophaga | Parulidae | Passeriformes | Aves |
| *Setophaga occidentalis* | Setophaga | Parulidae | Passeriformes | Aves |
| *Setophaga townsendi* | Setophaga | Parulidae | Passeriformes | Aves |
| *Sialia sialis* | Sialia | Turdidae | Passeriformes | Aves |
| *Spinus psaltria* | Spinus | Fringillidae | Passeriformes | Aves |
| *Sphyrapicus varius* | Sphyrapicus | Picidae | Piciformes | Aves |
| *Stelgidopteryx serripennis* | Stelgidopterix | Hirundinidae | Passeriformes | Aves |
| *Tilmatura dupontii* | Tilmatura | Trochilidae | Caprimulgiformes | Aves |
| *Pheugopedius felix* | Pheugopedius | Troglodytidae | Passeriformes | Aves |
| *Thryophilus sinaloa* | Thryophilus | Troglodytidae | Passeriformes | Aves |
| *Troglodytes aedon* | Troglodytes | Troglodytidae | Passeriformes | Aves |
| *Trogon elegans* | Trogon | Trogonidae | Trogoniformes | Aves |
| *Turdus assimilis* | Turdus | Turdidae | Passeriformes | Aves |
| *Turdus migratorius* | Turdus | Turdidae | Passeriformes | Aves |
| *Turdus rufopalliatus* | Turdus | Turdidae | Passeriformes | Aves |
| *Tyrannus verticalis* | Tyrannus | Tyrannidae | Passeriformes | Aves |
| *Vireo cassini* | Vireo | Vireonidae | Passeriformes | Aves |
| *Vireo flavoviridis* | Vireo | Vireonidae | Passeriformes | Aves |
| *Vireo hypochryseus* | Vireo | Vireonidae | Passeriformes | Aves |
| *Zenaida asiatica* | Zenaida | Columbidae | Columbiformes | Aves |
| *Zenaida macroura* | Zenaida | Columbidae | Columbiformes | Aves |
| *Didelphis virginiana* | Didelphis | Didelphidae | Didelphimorphia | Mammalia |
| *Sylvilagus cunicularius* | Sylvilagus | Leporidae | Lagomorpha | Mammalia |
| *Sylvilagus floridanus* | Sylvilagus | Leporidae | Lagomorpha | Mammalia |
| *Sciurus aureogaster* | Sciurus | Sciuridae | Rodentia | Mammalia |
| *Sciurus colliaei* | Sciurus | Sciuridae | Rodentia | Mammalia |
| *Sciurus nayaritensis* | Sciurus | Sciuridae | Rodentia | Mammalia |
| *Otospermophilus variegatus* | Otospermophilus | Sciuridae | Rodentia | Mammalia |
| *Herpailurus yagouaroundi* | Herpailurus | Felidae | Carnivora | Mammalia |
| *Leopardus pardalis* | Leopardus | Felidae | Carnivora | Mammalia |
| *Leopardus wiedii* | Leopardus | Felidae | Carnivora | Mammalia |
| *Lynx rufus* | Lynx | Felidae | Carnivora | Mammalia |
| *Puma concolor* | Puma | Felidae | Carnivora | Mammalia |
| *Panthera onca* | Panthera | Felidae | Carnivora | Mammalia |
| *Canis latrans* | Canis | Canidae | Carnivora | Mammalia |
| *Urocyon cinereoargenteus* | Urocyon | Canidae | Carnivora | Mammalia |
| *Conepatus leuconotus* | Conepatus | Mephitidae | Carnivora | Mammalia |
| *Mephitis macroura* | Mephitis | Mephitidae | Carnivora | Mammalia |
| *Spilogale angustifrons* | Spilogale | Mephitidae | Carnivora | Mammalia |
| *Dasypus novemcinctus* | Dasypus | Dasypodidae | Cingulata | Mammalia |
| *Lontra longicaudis* | Lontra | Mustelidae | Carnivora | Mammalia |
| *Mustela frenata* | Mustela | Mustelidae | Carnivora | Mammalia |
| *Bassariscus astutus* | Bassariscus | Procyonidae | Carnivora | Mammalia |
| *Nasua narica* | Nasua | Procyonidae | Carnivora | Mammalia |
| *Procyon lotor* | Procyon | Procyonidae | Carnivora | Mammalia |
| *Dicotyles tajacu* | Dicotyles | Tayassuidae | Artiodactyla | Mammalia |
| *Odocoileus virginianus* | Odocoileus | Cervidae | Artiodactyla | Mammalia |
| *Desmodus rotundus* | Desmodus | Phyllostomatidae | Chiroptera | Mammalia |
| *Micronycteris microtis* | Micronycteris | Phyllostomatidae | Chiroptera | Mammalia |
| *Anoura geoffroyi* | Anoura | Phyllostomatidae | Chiroptera | Mammalia |
| *Choeroniscus godmani* | Choeroniscus | Phyllostomatidae | Chiroptera | Mammalia |
| *Choeroniscus mexicana* | Choeroniscus | Phyllostomatidae | Chiroptera | Mammalia |
| *Artibeus jamaicensis* | Artibeus | Phyllostomatidae | Chiroptera | Mammalia |
| *Artibeus lituratus* | Artibeus | Phyllostomatidae | Chiroptera | Mammalia |
| *Chiroderma salvini* | Chiroderma | Phyllostomatidae | Chiroptera | Mammalia |
| *Dermanura tolteca* | Dermanura | Phyllostomatidae | Chiroptera | Mammalia |
| *Enchisthenes hartii* | Enchisthenes | Phyllostomatidae | Chiroptera | Mammalia |
| *Sturnira hondurensis* | Sturnira | Phyllostomatidae | Chiroptera | Mammalia |
| *Sturnira parvidens* | Sturnira | Phyllostomatidae | Chiroptera | Mammalia |
| *Leptpnycteris nivalis* | Leptonycteris | Phyllostomatidae | Chiroptera | Mammalia |
| *Glossophaga sp.* | Glossophaga | Phyllostomatidae | Chiroptera | Mammalia |
| *Pteronotus parnellii* | Pteronotus | Mormoopidae | Chiroptera | Mammalia |
| *Tadarida brasiliensis* | Tadarida | Molossidae | Chiroptera | Mammalia |
| *Myotis auriculus* | Myotis | Vespertilionidae | Chiroptera | Mammalia |
| *Myotis californicus* | Myotis | Vespertilionidae | Chiroptera | Mammalia |
| *Myotis fortidens* | Myotis | Vespertilionidae | Chiroptera | Mammalia |
| *Myotis thysanodes* | Myotis | Vespertilionidae | Chiroptera | Mammalia |
| *Myotis yumanensis* | Myotis | Vespertilionidae | Chiroptera | Mammalia |
| *Corynorhinus mexicanus* | Corynorhinus | Vespertilionidae | Chiroptera | Mammalia |
| *Eptesicus furinalis* | Eptesicus | Vespertilionidae | Chiroptera | Mammalia |
| *Eptesicus fuscus* | Eptesicus | Vespertilionidae | Chiroptera | Mammalia |
| *Lasiurus frantzii* | Lasiurus | Vespertilionidae | Chiroptera | Mammalia |
| *Lasiurus cinereus* | Lasiurus | Vespertilionidae | Chiroptera | Mammalia |
| *Lasiurus intermedius* | Lasiurus | Vespertilionidae | Chiroptera | Mammalia |
| *Lasiurus xanthinus* | Lasiurus | Vespertilionidae | Chiroptera | Mammalia |
| *Rhogeessa alleni* | Rhogeessa | Vespertilionidae | Chiroptera | Mammalia |
| *Rhogeessa gracilis* | Rhogeessa | Vespertilionidae | Chiroptera | Mammalia |
| *Rhogeessa parvula* | Rhogeessa | Vespertilionidae | Chiroptera | Mammalia |
| *Pappogeomys bulleri* | Pappogeomys | Geomyidae | Rodentia | Mammalia |
| *Heteromys pictus* | Heteromys | Heteromyidae | Rodentia | Mammalia |
| *Neotoma mexicana* | Neotoma | Cricetidae | Rodentia | Mammalia |
| *Sigmodon alleni* | Sigmodon | Cricetidae | Rodentia | Mammalia |
| *Sigmodon hispidus* | Sigmodon | Cricetidae | Rodentia | Mammalia |
| *Reithrodontomys megalotis* | Reithrodontomys | Cricetidae | Rodentia | Mammalia |
| *Reithrodontomys zacatecae* | Reithrodontomys | Cricetidae | Rodentia | Mammalia |
| *Reithrodontomys fulvescens* | Reithrodontomys | Cricetidae | Rodentia | Mammalia |
| *Baiomys musculus* | Baiomys | Cricetidae | Rodentia | Mammalia |
| *Peromyscus spicilegus* | Peromyscus | Cricetidae | Rodentia | Mammalia |
| *Peromyscus hylocetes* | Peromyscus | Cricetidae | Rodentia | Mammalia |
| *Peromyscus boylii* | Peromyscus | Cricetidae | Rodentia | Mammalia |
| *Peromyscus maniculatus* | Peromyscus | Cricetidae | Rodentia | Mammalia |
| *Peromyscus melanotis* | Peromyscus | Cricetidae | Rodentia | Mammalia |
| *Cryptotis parvus* | Cryptotis | Soricidae | Soricomorpha | Mammalia |
|  |  |  |  |  |
