# Supplementary material for: Taxonomic diversity of terrestrial vertebrates in west-central Mexico: Conservation from a multi-taxa perspective
Source: PLoS One. 2024 Oct 9;19(10):e0311770. doi: 10.1371/journal.pone.0311770 (PMC11463785; doi:10.1371/journal.pone.0311770)
Supplement: S3 Table — (DOCX) [file pone.0311770.s003.docx]

Supplementary material

Taxonomic diversity of terrestrial vertebrates in west-central Mexico: conservation from a multi-taxa perspective

Eliza Álvarez-Grzybowska^1,2^, Verónica Carolina Rosas-Espinoza^2^, Karen Elizabeth Peña-Joya^3^, Ana Luisa Santiago-Pérez^4^, Luis Ignacio Íñiguez-Dávalos^5^, Miguel Ángel Macías-Rodríguez^6^, Fabián Alejandro Rodríguez-Zaragoza^2*^

^1^ Doctorado en Biosistemática, Ecología y Manejo de Recursos Naturales y Agrícolas (BEMARENA), Centro Universitario de Ciencias Biológicas y Agropecuarias, Universidad de Guadalajara, Zapopan, Jalisco, México

^2^ Laboratorio de Ecología Molecular, Microbiología y Taxonomía (LEMITAX), Departamento de Ecología Aplicada, Centro Universitario de Ciencias Biológicas y Agropecuarias, Universidad de Guadalajara, Zaopan, Jalisco, México

^3^ Laboratorio de Ecología, Paisaje y Sociedad, Centro Universitario de la Costa, Universidad de Guadalajara, Puerto Vallarta, Jalisco, México

^4^ Departamento de Producción Forestal, Centro Universitario de Ciencias Biológicas y Agropecuarias, Universidad de Guadalajara, Zapopan, Jalisco, México

^5^ Departamento de Ecología y Recursos Naturales, Centro Universitario de la Costa Sur, Universidad de Guadalajara, Autlán de Navarro, Jalisco, México

^6^ Departamento de Ciencias Ambientales, Centro Universitario de Ciencias Biológicas y Agropecuarias, Universidad de Guadalajara, Zapopan 45200, Jalisco, México

*Corresponding author

E-mail: [fabian.rzaragoza@academicos.udg.mx](mailto:fabian.rzaragoza@academicos.udg.mx) (FARZ)

**Table S3.** **Exclusive richness in tropical and temperate environments and taxa present in all habitat types from the SQPA.**

|  | **Tropical  habitats** | **Temperate habitats** | **All habitats** |
| --- | --- | --- | --- |
| **Amphibians** |  |  |  |
| Species | 8 | 4 | 0 |
| Genera | 7 | 3 | 0 |
| Families | 5 | 3 | 0 |
| Orders | 0 | 1 | 0 |
| **Reptiles** |  |  |  |
| Species | 8 | 11 | 1 |
| Genera | 4 | 8 | 1 |
| Families | 2 | 2 | 1 |
| Orders | 0 | 0 | 0 |
| **Birds** |  |  |  |
| Species | 20 | 36 | 5 |
| Genera | 14 | 24 | 4 |
| Families | 8 | 8 | 4 |
| Orders | 3 | 3 | 3 |
| **Mammals** |  |  |  |
| Species | 7 | 20 | 14 |
| Genera | 6 | 13 | 13 |
| Families | 2 | 6 | 10 |
| Orders | 0 | 3 | 4 |
|  |  |  |  |
| Total | 43 | 71 | 20 |
