# Supplementary material for: Taxonomic diversity of terrestrial vertebrates in west-central Mexico: Conservation from a multi-taxa perspective
Source: PLoS One. 2024 Oct 9;19(10):e0311770. doi: 10.1371/journal.pone.0311770 (PMC11463785; doi:10.1371/journal.pone.0311770)
Supplement: S4 Table — Codes: TDF, tropical dry forest; GFTr, tropical gallery forest; OF, oak forest; MF, mixed forest; GFTe, temperate gallery forest. Bold numbers represent significant differences (p ≤ 0.05) based on 10,000 permutations. (DOCX) [file pone.0311770.s004.docx]

Supplementary material

Taxonomic diversity of terrestrial vertebrates in west-central Mexico: conservation from a multi-taxa perspective

Eliza Álvarez-Grzybowska^1,2^, Verónica Carolina Rosas-Espinoza^2^, Karen Elizabeth Peña-Joya^3^, Ana Luisa Santiago-Pérez^4^, Luis Ignacio Íñiguez-Dávalos^5^, Miguel Ángel Macías-Rodríguez^6^, Fabián Alejandro Rodríguez-Zaragoza^2*^

^1^ Doctorado en Biosistemática, Ecología y Manejo de Recursos Naturales y Agrícolas (BEMARENA), Centro Universitario de Ciencias Biológicas y Agropecuarias, Universidad de Guadalajara, Zapopan, Jalisco, México

^2^ Laboratorio de Ecología Molecular, Microbiología y Taxonomía (LEMITAX), Departamento de Ecología Aplicada, Centro Universitario de Ciencias Biológicas y Agropecuarias, Universidad de Guadalajara, Zaopan, Jalisco, México

^3^ Laboratorio de Ecología, Paisaje y Sociedad, Centro Universitario de la Costa, Universidad de Guadalajara, Puerto Vallarta, Jalisco, México

^4^ Departamento de Producción Forestal, Centro Universitario de Ciencias Biológicas y Agropecuarias, Universidad de Guadalajara, Zapopan, Jalisco, México

^5^ Departamento de Ecología y Recursos Naturales, Centro Universitario de la Costa Sur, Universidad de Guadalajara, Autlán de Navarro, Jalisco, México

^6^ Departamento de Ciencias Ambientales, Centro Universitario de Ciencias Biológicas y Agropecuarias, Universidad de Guadalajara, Zapopan 45200, Jalisco, México

*Corresponding author

E-mail: [fabian.rzaragoza@academicos.udg.mx](mailto:fabian.rzaragoza@academicos.udg.mx) (FARZ)

**Table S4.** **Results of the average taxonomic distinctiveness analyses** **overall and at level of taxonomic groups in the SQPA.** Codes: TDF, tropical dry forest; GFTr, tropical gallery forest; OF, oak forest; MF, mixed forest; GFTe, temperate gallery forest. Bold numbers represent significant differences (p ≤ 0.05) based on 10,000 permutations.

| **Spatial level** | **S** | $\boldsymbol{\Delta}$**^+^** | **p** | $\boldsymbol{\Lambda}$**^+^** | **p** |
| --- | --- | --- | --- | --- | --- |
| **Overall** |  |  |  |  |  |
| TDF | 123 | 88.1 | 0.1 | 278.0 | 0.941 |
| GFTr | 122 | 89.1 | 0.853 | 277.8 | 0.923 |
| OF | 117 | 88.9 | 0.743 | 277.0 | 0.955 |
| MF | 125 | 88.4 | 0.158 | 287.4 | 0.412 |
| GFTe | 102 | 86.4 | **0.004** | 337.8 | **0.002** |
|  |  |  |  |  |  |
| **Amphibians** |  |  |  |  |  |
| TDF | 5 | 60.0 | 1.0 | 0.0 | 0.148 |
| GFTr | 14 | 56.3 | **0.05** | 87.1 | **0.016** |
| OF | 5 | 68.0 | 0.452 | 36.0 | 0.092 |
| MF | 4 | 60.0 | 1.0 | 0.0 | 0.424 |
| GFTe | 5 | 72.0 | 0.078 | 96.0 | 0.953 |
|  |  |  |  |  |  |
| **Reptiles** |  |  |  |  |  |
| TDF | 8 | 55.7 | 0.757 | 153.1 | 0.923 |
| GFTr | 11 | 54.6 | 1.0 | 181.2 | 0.513 |
| OF | 12 | 54.6 | 1.0 | 152.1 | 1.0 |
| MF | 11 | 56.4 | 0.342 | 88.6 | 0.298 |
| OF | 12 | 54.6 | 1.0 | 152.1 | 1.0 |
| GFTe | 3 | 53.3 | 1.0 | 88.9 | 0.931 |
|  |  |  |  |  |  |
| **Birds** |  |  |  |  |  |
| TDF | 59 | 70.1 | 0.747 | 147.2 | 0.394 |
| GFTr | 62 | 70.5 | 0.503 | 142.5 | 0.082 |
| OF | 53 | 69.3 | 0.755 | 183.7 | **0.002** |
| MF | 54 | 68.2 | 0.224 | 180.6 | **0.01** |
| GFTe | 50 | 65.1 | **0.004** | 170.3 | 0.104 |
|  |  |  |  |  |  |
| **Mammals** |  |  |  |  |  |
| TDF | 51 | 71.6 | 0.951 | 199.8 | 0.342 |
| GFTr | 35 | 69.1 | **0.038** | 237.8 | 0.342 |
| OF | 47 | 72.8 | 0.094 | 181.9 | 0.054 |
| MF | 56 | 72.7 | **0.014** | 199.0 | 0.192 |
| GFTe | 44 | 71.8 | 0.883 | 229.5 | 0.372 |
