# Supplementary material for: Taxonomic diversity of terrestrial vertebrates in west-central Mexico: Conservation from a multi-taxa perspective
Source: PLoS One. 2024 Oct 9;19(10):e0311770. doi: 10.1371/journal.pone.0311770 (PMC11463785; doi:10.1371/journal.pone.0311770)
Supplement: S5 Table — Codes: β.3, turnover component; βrich, differences in richness component; TDF, tropical dry forest; GFTr tropical gallery forest; OF, oak forest; MF, mixed forest; GFTe, temperate gallery forest. Bold numbers represent values greater than 0.5 of taxonomic dissimilarity. (DOCX) [file pone.0311770.s005.docx]

Supplementary material

Taxonomic diversity of terrestrial vertebrates in west-central Mexico: conservation from a multi-taxa perspective

Eliza Álvarez-Grzybowska^1,2^, Verónica Carolina Rosas-Espinoza^2^, Karen Elizabeth Peña-Joya^3^, Ana Luisa Santiago-Pérez^4^, Luis Ignacio Íñiguez-Dávalos^5^, Miguel Ángel Macías-Rodríguez^6^, Fabián Alejandro Rodríguez-Zaragoza^2*^

^1^ Doctorado en Biosistemática, Ecología y Manejo de Recursos Naturales y Agrícolas (BEMARENA), Centro Universitario de Ciencias Biológicas y Agropecuarias, Universidad de Guadalajara, Zapopan, Jalisco, México

^2^ Laboratorio de Ecología Molecular, Microbiología y Taxonomía (LEMITAX), Departamento de Ecología Aplicada, Centro Universitario de Ciencias Biológicas y Agropecuarias, Universidad de Guadalajara, Zaopan, Jalisco, México

^3^ Laboratorio de Ecología, Paisaje y Sociedad, Centro Universitario de la Costa, Universidad de Guadalajara, Puerto Vallarta, Jalisco, México

^4^ Departamento de Producción Forestal, Centro Universitario de Ciencias Biológicas y Agropecuarias, Universidad de Guadalajara, Zapopan, Jalisco, México

^5^ Departamento de Ecología y Recursos Naturales, Centro Universitario de la Costa Sur, Universidad de Guadalajara, Autlán de Navarro, Jalisco, México

^6^ Departamento de Ciencias Ambientales, Centro Universitario de Ciencias Biológicas y Agropecuarias, Universidad de Guadalajara, Zapopan 45200, Jalisco, México

*Corresponding author

E-mail: [fabian.rzaragoza@academicos.udg.mx](mailto:fabian.rzaragoza@academicos.udg.mx) (FARZ)

**Table S5.** **Taxonomic beta diversity additive partitioning results of terrestrial vertebrates between vegetation types.** Codes: *β_.3_*, turnover component; *β_rich_*, differences in richness component; TDF, tropical dry forest; MF, mixed forest; OF, oak forest; GFTe, temperate gallery forest; GFTr, tropical gallery forest. Bold numbers represent values greater than 0.5 of taxonomic dissimilarity.

| **Comparisons** | | **Overall** | | | **Amphibians** | | | **Reptiles** | | | **Birds** | | | **Mammals** | | |
| --- | --- | --- | --- | --- | --- | --- | --- | --- | --- | --- | --- | --- | --- | --- | --- | --- |
|  |  | *β_.3_* | *β_rich_* | *β_CC_* | *β_.3_* | *β_rich_* | *β_CC_* | *β_.3_* | *β_rich_* | *β_CC_* | *β_.3_* | *β_rich_* | *β_CC_* | *β_.3_* | *β_rich_* | *β_CC_* |
| Paired-beta | TDF-GFTr | 0.29 | 0.02 | 0.31 | 0.00 | 0.50 | **0.50** | 0.27 | 0.13 | 0.40 | 0.20 | 0.05 | 0.25 | 0.08 | 0.29 | 0.37 |
|  | OF-TDF | 0.50 | 0.06 | **0.56** | 0.64 | 0.04 | **0.68** | 0.58 | 0.13 | **0.71** | 0.64 | 0.10 | **0.74** | 0.20 | 0.05 | 0.25 |
|  | TDF-MF | 0.45 | 0.03 | 0.48 | 0.21 | 0.16 | 0.37 | 0.53 | 0.16 | **0.69** | 0.46 | 0.10 | **0.56** | 0.32 | 0.04 | 0.36 |
|  | TDF-GFTe | 0.44 | 0.16 | **0.60** | 0.74 | 0.00 | **0.74** | 0.37 | 0.44 | **0.81** | 0.54 | 0.15 | **0.69** | 0.32 | 0.15 | 0.47 |
|  | OF-GFTr | 0.59 | 0.04 | **0.63** | 0.38 | 0.43 | **0.81** | 0.68 | 0.02 | **0.70** | 0.53 | 0.14 | **0.67** | 0.29 | 0.22 | **0.51** |
|  | GFTr-MF | 0.58 | 0.01 | **0.59** | 0.06 | 0.57 | **0.63** | 0.70 | 0.05 | **0.75** | 0.47 | 0.13 | **0.60** | 0.26 | 0.29 | **0.55** |
|  | GFTr-GFTe | 0.44 | 0.15 | **0.59** | 0.38 | 0.40 | **0.78** | 0.27 | 0.53 | **0.80** | 0.43 | 0.19 | **0.62** | 0.32 | 0.12 | 0.44 |
|  | OF-MF | 0.44 | 0.03 | 0.47 | 0.61 | 0.09 | **0.70** | 0.40 | 0.03 | 0.43 | 0.63 | 0.01 | **0.64** | 0.16 | 0.10 | 0.26 |
|  | OF-GFTe | 0.32 | 0.12 | 0.44 | 0.45 | 0.05 | **0.50** | 0.00 | 0.63 | **0.63** | 0.35 | 0.06 | 0.41 | 0.32 | 0.11 | 0.43 |
|  | GFTe-MF | 0.38 | 0.14 | **0.52** | 0.58 | 0.13 | **0.71** | 0.13 | 0.60 | **0.73** | 0.60 | 0.06 | **0.66** | 0.06 | 0.21 | 0.27 |
| Multi-beta | | 0.44 | 0.08 | **0.52** | 0.41 | 0.24 | **0.65** | 0.39 | 0.27 | **0.66** | 0.49 | 0.10 | **0.59** | 0.23 | 0.16 | 0.39 |
